# Short versus long silver nanowires: a comparison of *in vivo* pulmonary effects post instillation

**DOI:** 10.1186/s12989-014-0052-6

**Published:** 2014-10-08

**Authors:** Rona M Silva, Jingyi Xu, Clare Saiki, Donald S Anderson, Lisa M Franzi, Chris D Vulpe, Benjamin Gilbert, Laura S Van Winkle, Kent E Pinkerton

**Affiliations:** Center for Health and the Environment, University of California, One Shields Avenue, Davis, CA 95616 USA; Department of Endocrinology, Affiliated Zhongshan Hospital of Dalian University, Dalian, Liaoning 116001 China; Department of Pulmonary Medicine, School of Medicine, University of California, Davis, CA 95616 USA; Department of Nutritional Science and Toxicology, University of California, Berkeley, CA 94720 USA; Geochemistry Department, Berkeley Nanogeoscience Center, University of California, Berkeley, CA 94720 USA

**Keywords:** Silver nanowires, Pulmonary toxicity, Macrophage, Histopathology, *In vivo*, Intratracheal instillation

## Abstract

**Background:**

Silver nanowires (Ag NWs) are increasingly being used to produce touchscreens for smart phones and computers. When applied in a thin film over a plastic substrate, Ag NWs create a transparent, highly-conductive network of fibers enabling the touch interface between consumers and their electronics. Large-scale application methods utilize techniques whereby Ag NW suspensions are deposited onto substrates via droplets. Aerosolized droplets increase risk of occupational Ag NW exposure. Currently, there are few published studies on Ag NW exposure-related health effects. Concerns have risen about the potential for greater toxicity from exposure to high-aspect ratio nanomaterials compared to their non-fibrous counterparts. This study examines whether Ag NWs of varying lengths affect biological responses and silver distribution within the lungs at different time-points.

**Methods:**

Two different sizes of Ag NWs (2 μm [S-Ag NWs] and 20 μm [L-Ag NWs]) were tested. Male, Sprague-Dawley rats were intratracheally instilled with Ag NWs (0, 0.1, 0.5, or 1.0 mg/kg). Broncho-alveolar lavage fluid (BALF) and lung tissues were obtained at 1, 7, and 21 days post exposure for analysis of BAL total cells, cell differentials, and total protein as well as tissue pathology and silver distribution.

**Results and conclusions:**

The two highest doses produced significant increases in BAL endpoints. At Day 1, Ag NWs increased total cells, inflammatory polymorphonuclear cells (PMNs), and total protein. PMNs persisted for both Ag NW types at Day 7, though not significantly so, and by Day 21, PMNs appeared in line with sham control values. Striking histopathological features associated with Ag NWs included 1) a strong influx of eosinophils at Days 1 and 7; and 2) formation of Langhans and foreign body giant cells at Days 7 and 21. Epithelial sloughing in the terminal bronchioles (TB) and cellular exudate in alveolar regions were also common. By Day 21, Ag NWs were primarily enclosed in granulomas or surrounded by numerous macrophages in the TB-alveolar duct junction. These findings suggest short and long Ag NWs produce pulmonary toxicity; thus, further research into exposure-related health effects and possible exposure scenarios are necessary to ensure human safety as Ag NW demand increases.

**Electronic supplementary material:**

The online version of this article (doi:10.1186/s12989-014-0052-6) contains supplementary material, which is available to authorized users.

## Background

Many people unknowingly use silver nanowire (Ag NW) technology on a daily basis. Ag NWs are increasingly being promoted and/or applied in the production of flexible and lightweight touchscreens used for smart phones, GPS and gaming systems, tablets, and computers. When applied in a thin film over a plastic substrate, Ag NWs create a transparent and highly conductive network of fibers that enables the touch interface between consumers and their electronics. According to various forecasts, the market for transparent conductors is expected to grow to between $2 billion and $7 billion in the next four years alone [1,2].

The most promising, upwardly-scalable Ag NW application methods utilize techniques whereby nanowire suspensions are deposited onto substrates via droplets. Aerosolized droplets pose a risk of occupational exposure via the eyes, skin, and lungs when proper protective equipment and ventilation are not employed. Currently, the vast majority of published, peer-reviewed, nano-silver research focuses on Ag nanoparticles (Ag NPs), or the production and application of Ag NWs. Only a meager proportion investigates Ag NW-related health effects, and no studies have looked at actual human exposures to Ag NWs. In fact, literature searches of two large peer-reviewed journal databases (Web of Science and Pubmed) found less than 15 journal articles concerning *in vitro* [3-9] and/or *in vivo* [10-13] responses to Ag NW exposure. Of the *in vivo* work, only two of the studies were done in mammals [12,13].

Previous *in vivo* studies have shown that Ag NPs produce inflammatory lung lesions, and decrements in lung function in Sprague-Dawley (SD) rats during a 90-day inhalation exposure period [14], and mild sub-chronic fibrogenic effects in C57Bl/6 mice upon oropharyngeal aspiration [15]. Research suggests that Ag NP exposure also results in perivascular collagen deposition [15], and changes in red blood cell morphology with decreased deformability and aggregation, which could alter hemodynamics [5]. Silver ions (Ag^+^) shed from Ag NPs have been shown to produce toxic responses correlated to their rate of release [15-17], and rate of Ag^+^ release is associated with Ag NP size and surface area as well as ambient conditions (e.g. oxygen and light exposure) [5,15,17]. Considering 1) the relatively higher toxicity of high-aspect ratio nanomaterials (HARN) to their non-fibrous, particulate counterparts; 2) the minimally-studied toxic effects of Ag NW exposures; and 3) the inadequate data on real-life Ag NW exposure concentrations and resulting health effects, the study described herein aimed to discern how Ag NWs of varying lengths affect biological responses and silver distribution throughout the lungs at different time-points.

Schinwald and colleagues have defined a clear threshold length (≥5 μm) at which HARN produce pathogenicity in the pleural space [9]. They thoroughly demonstrated that the threshold length at which frustrated phagocytosis occurs *in vivo* (≥10 μm) is lower than that *in vitro* (≥14 μm) due to the abnormal experimental conditions inherent in the latter [12]. However, their research focuses primarily on responses in the pleural space upon intra-pleural instillation of Ag NWs. The aim of the unique research presented herein is to expand upon previous findings by assessing Ag NW distribution, and responses by various cell types, in multiple regions of the lung at several time-points, after intratracheal instillation (IT).

Two types of polyvinylpyrrolidone (PVP)-coated Ag NWs were tested: 2 μm S-Ag NWs and 20 μm L-Ag NWs. PVP is often used as a coordinating agent during Ag NW synthesis to ensure wire formation, and it maintains nanowire dispersion in suspension for even distribution of Ag NWs in thin films and other matrices. Male SD rats were administered Ag NWs (0, 0.1, 0.5, or 1.0 mg/kg) via IT. Bronchoalveolar lavage fluid (BALF) and lung tissues were obtained for analysis at 1, 7, and 21 days post exposure. Results confirm S- and L-Ag NWs produce pulmonary toxicity, and that at Day 21 post IT, silver is still present in the lungs.

## Results

The physicochemical characterization of S- and L-Ag NWs is summarized in Table 1.

**Table 1:** **Physicochemical characterization of Ag NWs**

**Quality**	**Technique**	**S-Ag NWs**	**L-Ag NWs**
Mean length (μm)	TEM	2.0 ± 0.6	20.8 ± 10.8
Mean width (nm)	TEM	33.1 ± 5.7	64.7 ± 14.1
Mean aspect ratio	TEM	62.1	321.4
[Ag (0)] (mg/mL)	ICP-MS	3.38 ± 0.24	3.40 ± 0.12
[Ag +] (mg/mL)	ICP-MS	0.01	0.008

### Animal weight

Animal weight was unaffected by IT of Ag NWs. For animals exhibiting inflammation, there was no correlation between weight change following IT and subsequent degree of inflammation post exposure.

### BALF

**Figure 1:**
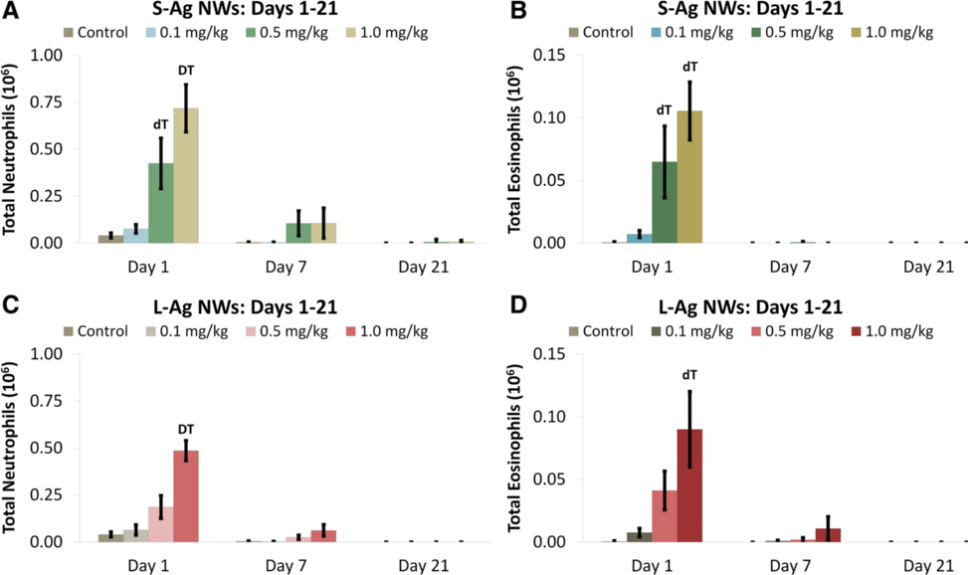
**Ag NWs produce acutely significant BALF neutrophilia and eosinophilia.** Graphs show absolute numbers of neutrophils (left) and eosinophils (right) on Days 1-21 post IT of S-Ag NWs **(A-B)**, or L-Ag NWs **(C-D)**. Scales on left and right panels are different. Results are from ANOVA considering dose, time, and particle length at *p* < 0.05. “D” corresponds specifically to differences between groups (in the same panel, sacrificed on the same day) given 1.0 mg/kg Ag NWs versus any other dose; while, “d” indicates differences from the sham control and 0.01 mg/kg groups only. “T” indicates differences between animals (in the same panel, given the same treatment) sacrificed on Day 1 versus Days 7 and 21; while, “t” indicates a difference from Day 21 only.

**Figure 2:**
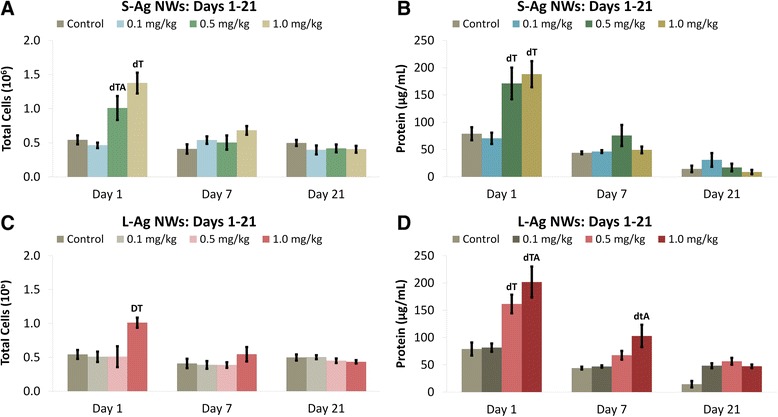
**Ag NWs increase total BALF cells and protein.** Graphs show absolute numbers of total cells (left), and proteins (right) in BALF on Days 1-21 post IT of S-Ag NWs **(A-B)**, or L-Ag NWs **(C-D)**. Results are from ANOVA considering dose, time, and particle length at *p* < 0.05. “D” corresponds specifically to differences between groups (in the same panel, sacrificed on the same day) given 1.0 mg/kg Ag NWs versus any other dose; while, “d” indicates differences from the sham control and 0.01 mg/kg groups only. “T” indicates differences between animals (in the same panel, given the same treatment) sacrificed on Day 1 versus Days 7 and 21; while, “t” indicates difference from Day 21 only. “A” indicates a significant difference between groups (in different panels) given the same dose of different Ag NW types (e.g. S- versus L-Ag NWs), but sacrificed on the same day.

Particle length, dose, and/or time post IT significantly influenced the numbers of BAL cells recovered (Figures 1 and 2, Additional file 1: Tables S1-S3). Ag NW instillation of the 0.5 and 1.0 mg/kg doses produced significant increases in neutrophil (Figure 1A & C) and eosinophil (Figure 1B & D) [polymorphonuclear cell (PMN)] numbers over sham controls at Day 1. Results suggested that Ag NW length had a marginal effect on the number of BALF neutrophils recovered post instillation [i.e. IT of S-Ag NWs produced more BALF neutrophils than L-Ag NWs when using particle mass (0, 0.1, 0.5, and 1.0 mg/kg bodyweight) dose metrics (Additional file 1: Tables S1-S2)]. However, it is possible that higher neutrophil numbers were observed 1 day post S-Ag NW versus L-Ag NW IT simply because of the greater numbers of particles delivered in the former versus the latter (Table 2). By Days 7 and 21 (Figure 1), there were no significant differences in neutrophil and/or eosinophil numbers between animals instilled with Ag NWs versus sham control. Findings are consistent with previous reports of pulmonary eosinophilic recruitment upon exposure to metal nanomaterials [18].

**Table 2:** **Instilled doses per rat**

**Ag NW type**	**Instilled Ag NW dose (mg/kg)**	*Average Ag NW mass instilled (mg)	*Average number of Ag NWs instilled (10 ^**9**^ **)**
**S-AgNWs**	0.1	0.035	1.94
	0.5	0.175	9.68
	1.0	0.350	19.37
**L-AgNWs**	0.1	0.035	0.05
	0.5	0.175	0.24
	1.0	0.350	0.49

“*” indicates calculations based upon an average 350 g rat.

Instillation of 0.5 mg/kg of S-Ag NWs, 1.0 mg/kg of S-Ag NWs, or 1.0 mg/kg of L- Ag NWs produced significant increases in total BAL cells at Day 1 in comparison to sham control (Figure 2A & C). S-Ag NWs produced more cell numbers than L-Ag NWs (Figure 2A) at Day 1 (only). By Days 7 and 21, total BAL cells were similar to control values for both S- and L-Ag NW-instilled animals. Results suggest that though S-Ag NWs produced significantly more cells at Day 1 post IT than L-Ag NWs, the inflammation was quickly resolved for both Ag NW lengths.

Similar to BAL cells, BALF protein was significantly increased at the two highest doses (0.5 or 1.0 mg/kg Ag NWs) compared to the control at Day 1 (Figure 2B & D). However, L-Ag NWs produced more protein than S-Ag NWs at the highest dose (Figure 2D). At Day 7, total protein decreased to control levels for all animals except those instilled with 1.0 mg/kg of L-Ag NWs, which had slight, but significantly elevated protein levels. By Day 21, BALF protein levels were comparable to control for all Ag NW instilled animals.

**Figure 3:**
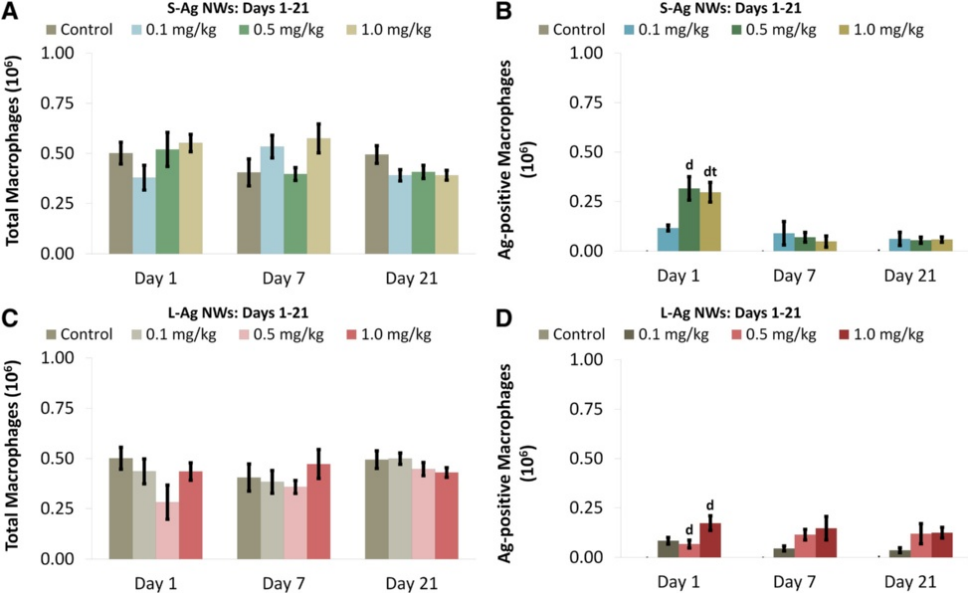
**Macrophage clearance of Ag NW.** Graph shows absolute numbers of macrophages (left), and silver-positive macrophages (right) in BALF on Days 1-21 post IT of S-Ag NWs **(A-B)**, or L-Ag NWs **(C-D)**. Results are from ANOVA considering dose, time, and particle length at *p* < 0.05. “d” indicates a significant difference from sham control group (in the same panel, sacrificed on the same day). “t” indicates a significant difference between animals (in the same panel, given the same treatment), but sacrificed on Day 1 versus Day 7.

**Figure 4:**
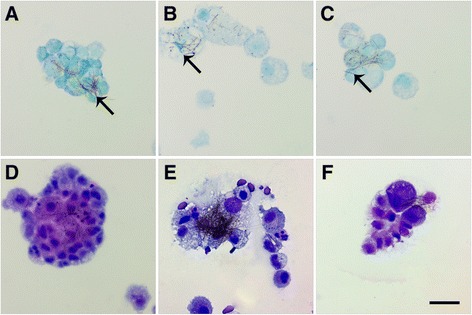
**L-Ag NWs produce frustrated phagocytosis.** Panels are Brightfield microscopy images of cells recovered from rat BALF at Days 1 (left), 7 (middle), and 21 (right) after a single instillation (1.0 mg/kg ) of L-Ag NWs (indicated by black arrows). BAL cells were stained using autometallography with a toluidine blue counter-stain **(A-C)**, or Diff Qwik® **(D-F)**. Images from slides stained with autometallography and Diff Qwik® are presented to enable the reader greater identification of Ag NW and cell types, respectively. Scale bar is 25 μm.

**Figure 5:**
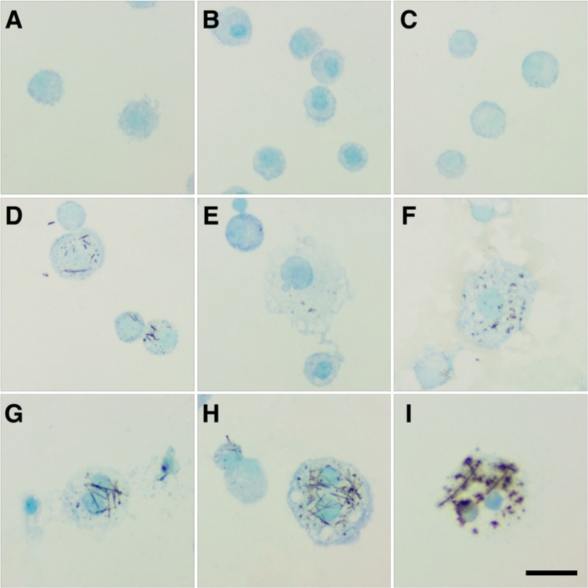
**Ag NWs produce giant foreign body macrophages in BALF.** Cells recovered from BALF at Days 1 (left), 7 (middle), and 21 (right) post exposure to sham control **(A-C)**, S-Ag NWs **(D-F)**, or L-Ag NWs **(G-I)**. All panels are Brightfield microscopy images of cells from rats given a single instillation of sham control or Ag NW suspension at 1.0 mL/kg. BAL cells were stained with autometallography and toluidine blue counter-stain. Arrows indicate Ag NWs. Scale bar is 25 μm.

There were no significant changes in the total number of BAL macrophages (Mφ) with exposure to Ag NWs (Figure 3A & C). However, quantification of Ag-positive Mφ confirmed that silver was present in Mφ through Day 21 (Figure 3B & D, Additional file 1: Figure S2). The two highest doses of both Ag NW lengths produced significant numbers of Ag-positive Mφ at Day 1 that then decreased temporally. The exact chemical state of the silver contained within Mφ was not determined herein, but there did appear to be several silver constituents present. Ag NWs were most commonly identifiable by their fibrous, particulate appearance and focal black-brown hues when stained using autometallography (Figure 4). However, some Mφ also had a diffuse brown coloration (Figure 5I, Additional file 1: Figure S2) in addition to Ag NWs, which was distinct from the faint toluidine blue counter-stain. This was observed on Days 1 and 7 for S-Ag NWs (Additional file 1: Figure S2A & B, respectively), and Days 1 and 21 for L-Ag NWs (Additional file 1: Figure S2C & D, respectively).

Though only L-Ag NW exposure produced bridges between multiple Mφ recovered in BALF (Figure 4), which resulted in frustrated phagocytosis of the nanowires throughout the course of the study, both S- and L-Ag NWs produced enlarged mono-/multi-nucleated Mφ, markers of foreign body responses, with varying degrees of vacuolization (Figure 5). Results are consistent with research suggesting that if foreign material is not removed within two-four weeks [19], Mφ begin fusing together, creating multi-nucleated foreign body giant cells (FBGCs) to aid in elimination. Macrophages were also observed with features of necrosis [e.g. swelling (Figure 5D-I, S1C-F), breakdown of the plasma membrane (Figure 5E-G & I, Additional file 1: Figure S1E), loss of intracellular contents (Figure 4E)] and apoptosis [e.g. membrane blebbing (Additional file 1: Figure S1C & F)] at all time-points after both L- and S-Ag NW exposure. By Day 21, S-Ag NWs sometimes appeared spherical, and many of the L-Ag NWs were fully enclosed by individual Mφ (Figure 5). Given the initial S-Ag NW and L-Ag NW lengths (2 μm and 20 μm, respectively), the results are consistent with research [12,20], which points to a ≥10 μm threshold for frustrated phagocytosis, and confirm that completely internalized Ag NWs can still produce inflammatory responses.

### Histopathology

Histopathological responses were scored using a semi-quantitative rubric that evaluated alveolitis, bronchiolitis, perivascular, particle-associated, and pleural inflammation (Additional file 1: Table S4, Figures S3-S4). Scores ranged from 0 (no histological response) to 3 (marked histological response). Inter-rater reliability for semi-quantitative histopathology scoring ranged from Kappa = 0.50 - 0.55, corresponding to moderate agreement with respect to exact matches; however, precision between raters was actually quite high. Of all the scores, approximately 63% were exact matches, and 37% were adjacent scores (±1).

**Figure 6:**
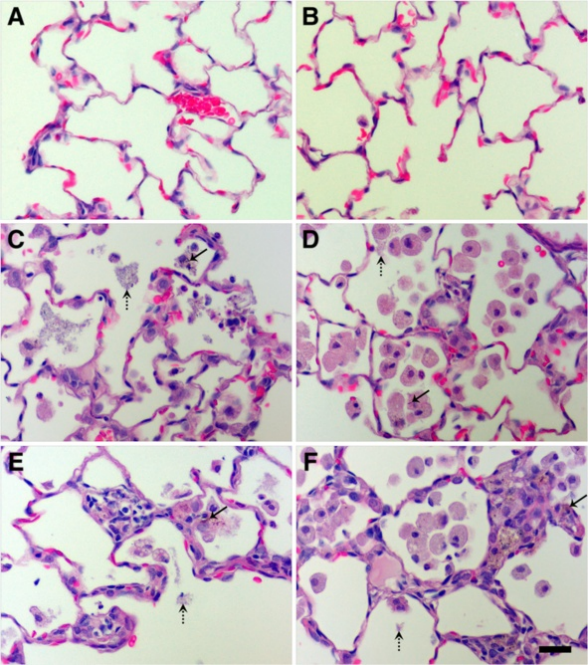
**Ag NW produced cellular exudate and irregular macrophages at Days 7 and 21.** H & E-stained tissue sections recovered at 7 (left) and 21 days (right) post exposure to sham control **(A-B)**, S-Ag NWs **(C-D)** or L-Ag NWs **(E-F)**. All panels are Brightfield microscopy images of representative tissues from rats instilled with a single 1.0 ml/kg dose. Solid arrows indicate Ag NWs, and broken arrows indicate cellular exudate. Scale bar is 25 μm.

**Figure 7:**
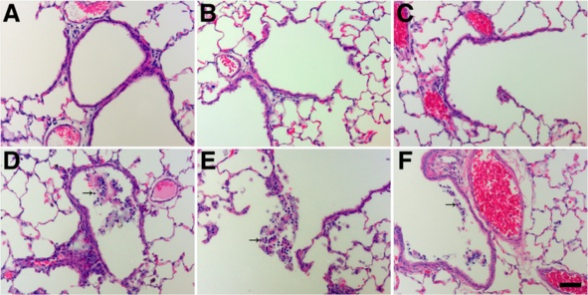
**Ag NW instillation produced epithelial sloughing in bronchiolar regions.** Panels are Brightfield microscopy images of the most severe responses in H & E-stained tissue sections, from rats instilled with a single dose of 1.0 ml/kg sham control **(A, B, C)**, L-Ag NWs **(D-E)**, or S-Ag NWs **(F)**. Images show tissues at Days 1, 7, and 21 days post exposure to sham control (A-C, respectively), Days 1 and 7 post exposure to L-Ag NWs (C & D, respectively), and Day 21 post exposure to S-Ag NWs **(F)**. Solid arrows indicate sloughed epithelial cells. Scale bar is 50 μm.

Overall, histological scores revealed that only instillation of 0.5 or 1.0 mg/kg S- or L-Ag NWs produced significant inflammation, and responses to S-Ag NWs appeared to worsen significantly with time (Additional file 1: Tables S5-S6, Figure S5). However, there were no significant differences in the severity of the inflammation observed upon instillation of S- versus L-Ag NWs irrespective of the dose, time post instillation, and/or histopathological endpoint. Findings (summarized in Table 3) included cellular exudate (consistent with cell lysis) and large/irregular Mφ in alveolar regions (Figure 6), and epithelial sloughing in the bronchiolar regions (Figure 7), in animals instilled with mid/high doses of either Ag NW type (but not sham control). Epithelial sloughing generally occurred at Days 1 and 7 for L-Ag NWs (Figure 7D and E, respectively) and Day 21 for S-Ag NWs (Figure 7F). Sloughing and cellular exudate were not common in control animals (Figures 6A-B, and 7A-C).

**Table 3:** **Summary of histopathology findings**

**Responses observed in lung tissues**	**S-AgNWs**			**L-AgNWs**		
	**Day 1**	**Day 7**	**Day 21**	**Day 1**	**Day 7**	**Day 21**
Cellular Exudate in Alveoli		X	X		X	X
Epithelial Sloughing			X	X	X	
Langhans Cells in Tissues		X	X			
Tissue Granulomas			X		X	X
*Alveolar Inflammation				X	X	
*Bronchiolar Inflammation					X	
*Particle- Associated Inflammation	X	X	X	X	X	X

“*” indicates that results were confirmed by statistical comparisons to control groups at a *p* value ≤ 0.05.

**Figure 8:**
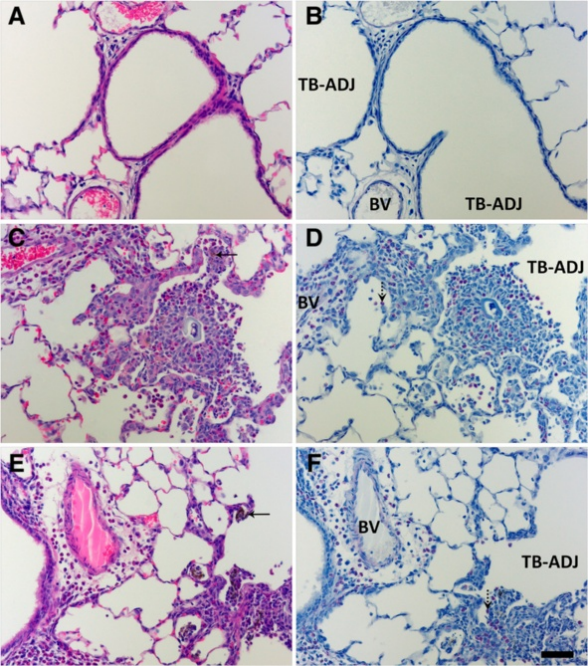
**At Day 1, Ag NW instillation produced marked inflammation in regions adjacent to particle agglomerates.** Images are from serial tissue sections stained with H & E (left) or CEM (right) stains, and recovered at 1 day post exposure to sham control **(A-B)**, S-Ag NWs **(C-D)**, or L-Ag NWs **(E-F)** Panels are Brightfield microscopy images of tissues from rats instilled with a single 0.5 ml/kg dose of sham control or Ag NWs (indicated by solid arrows). Broken arrows indicate eosinophils, which are bright pink in H & E and CEM panels. BV = blood vessel, and TB-ADJ = terminal bronchiole-alveolar duct junction. Scale bar is 50 μm.

**Figure 9:**
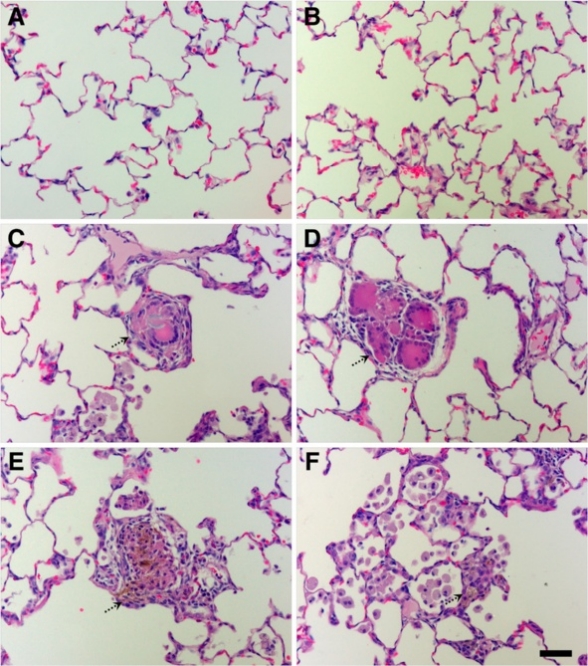
**Ag NWs produced foreign body reactions at Days 7 & 21 post instillation.** H & E-stained tissue sections recovered at 7 **(A, C, & E)** or 21 **(B, D, F)** days post exposure to sham control **(A-B)**, S-Ag NWs **(C-D)**, or L-Ag NWs **(E-F)**. All panels are Brightfield microscopy images of representative tissues from rats instilled with a single dose of sham control or Ag NWs at 0.5 ml/kg (Day 7), or 1.0 ml/kg (Day 21). Broken arrows indicate multi-nucleated Langhans cells **(C-D)**, or granulomas **(E-F)**. Scale bar is 50 μm.

Particle-associated inflammation, generally defined as increased cellularity adjacent to Ag NW agglomerates, was commonly seen post instillation of S- or L-Ag NWs with the 0.5 or 1.0 mg/kg doses (Figure 8, Additional file 1: Figure S5). At Day 1, particle agglomerates were often found near terminal bronchiole-alveolar duct junctions (TB-ADJs) and blood vessels (Figure 8C & E) accompanied by strong eosinophilic influx into the lungs, which was confirmed by combined eosinophil/mast cell (CEM) staining (Figure 8B, D, & F). Though Ag NW length did not appear to be a significant factor with respect to the severity of inflammation noted, it did influence the type of responses observed post instillation. At Day 7, mid/high Ag NW doses produced multi-nucleated foreign body Langhans cells in response to S-Ag NWs (Figure 9C), or granulomatous reactions around L-Ag NWs (Figure 9E). Though both Langhans cells and FBGC granulomas are formed by epithelioid cells (Mφ), the former is distinguishable by characteristic circular or horseshoe arrangements of cell nuclei in contrast to granulomas which exhibit less organized cell aggregates. The pattern of inflammation noted on Day 7 continued to Day 21, and though granulomas were observed for both Ag NW types, Langhans cells were only noted for S-Ag NWs (Figure 9D).

**Figure 10:**
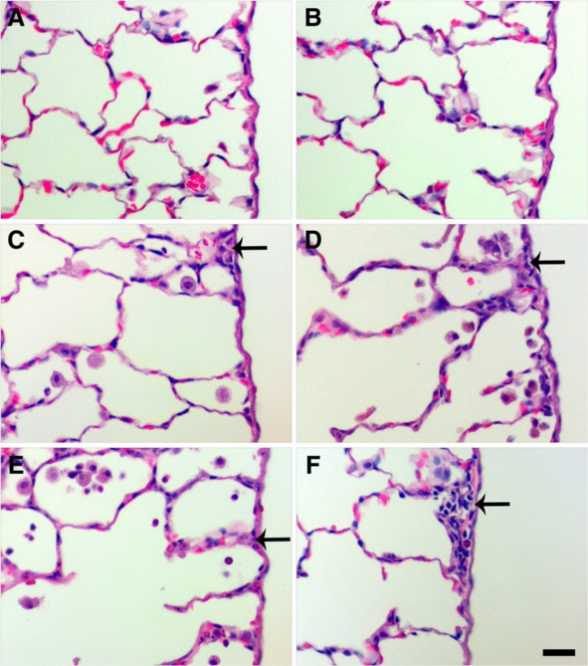
**Ag NW-induced pleural inflammation.** Images are H & E-stained tissue sections recovered at Days 1 (left) and 7 (right) post exposure to sham control **(A-B)**, S-Ag NWs **(C-D)**, or L-Ag NWs **(E-F)**. Panels are Brightfield microscopy images of responses in tissues from rats instilled with a single 1.0 ml/kg dose of sham control or Ag NWs. Solid arrows indicate thickened sub-pleural tissue. Scale bar is 25 μm.

Slight thickening of the sub-pleural connective tissue and a fair number of Mφ in the adjacent sub-pleural alveoli were noted at Days 1 and 7 (Figure 10). At Day 21 post exposure to L-Ag NWs (but not S-Ag NWs), mesothelial cells and/or pleural Mφ were observed clearly protruding from the pleural lining into the pleural space (Additional file 1: Figure S6). Groups of cells appeared in short, intermittent stretches in concave, flat, and convex regions along the pleura. These results are consistent with previous reports of pleural Mφ increases resulting from particulate exposure and parenchymal inflammation [21].

As Mφ attempt to phagocytose foreign materials, they produce numerous cytokines to stimulate the healing process. One such cytokine, tumor necrosis factor alpha (TNF-α), is produced by activated Mφ and cells in the alveolar and bronchiolar epithelium at early time-points during a pulmonary foreign-body reaction. TNF-α induces inflammation and apoptotic cell death. Another cytokine, transforming growth factor beta (TGF-β), which mediates cellular proliferation and differentiation, is associated with fibrotic responses to foreign materials.

**Figure 11:**
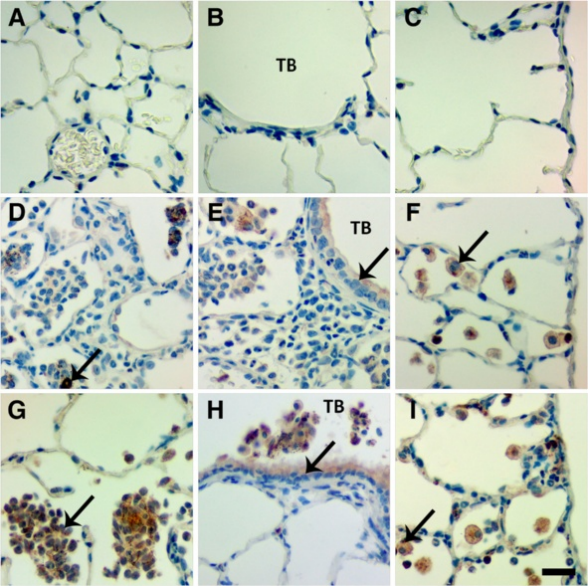
**Ag NWs produce TNF-α-positive cells at Day 1.** Tissue sections recovered at 1 day post instillation of sham control **(A-C)**, S-Ag NWs **(D-F)** or L-Ag NWs **(G-I)**, and stained with TNF-α immunohistochemical stain. Panels are Brightfield microscopy images of responses in tissues from rats instilled with a single dose of 1.0 mg/kg Ag NWs. Brown cells (indicated by arrows) in the alveoli (left), terminal bronchioles [TB (middle)], and pleural region (right) are TNF-α-positive. Scale bar is 25 μm.

**Figure 12:**
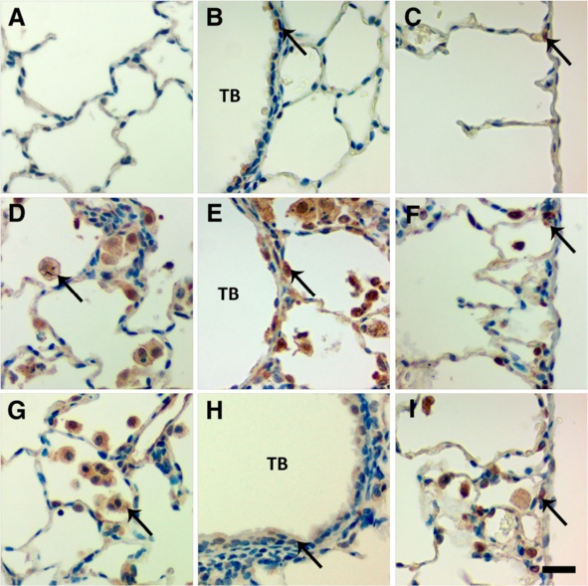
**Ag NWs produce TGF-β-positive cells at Day 7.** Tissue sections recovered at 7 days post instillation of sham control **(A-C)**, S-Ag NWs **(D-F)** or L-Ag NWs **(G-I)**, and stained with TGF-β immunohistochemical stain. Panels are Brightfield microscopy images of responses in tissues from rats instilled with a single dose of 1.0 mg/kg Ag NWs. Brown cells (indicated by arrows) in the alveoli (left), terminal bronchioles [TB (middle)], and pleural region (right) are TGF-β-positive. Scale bar is 25 μm.

**Figure 13:**
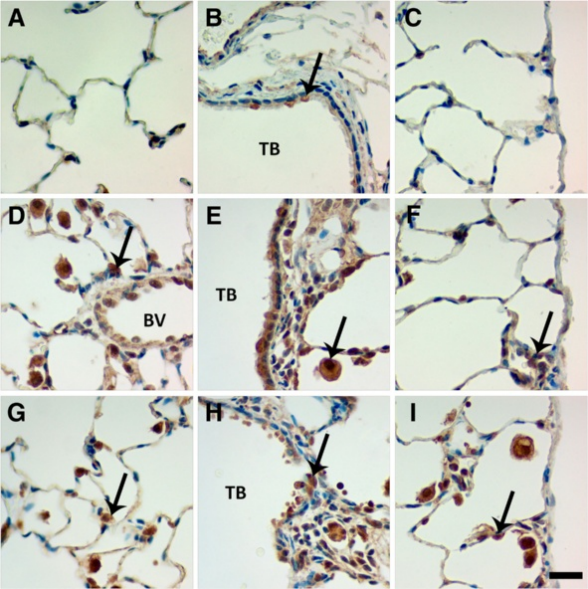
**TGF-β-positive cells are present at Day 21.** Tissue sections recovered at 21 days post instillation of sham control **(A-C)**, S-Ag NWs **(D-F)** or L-Ag NWs **(G-I)**, and stained with TGF-β immunohistochemical stain. Panels are Brightfield microscopy images of responses in tissues from rats instilled with a single dose of 1.0 mg/kg Ag NWs. Brown cells (indicated by arrows) in the alveoli (left), terminal bronchioles [TB (middle)], and pleural region (right) are TGF-β-positive. BV = blood vessel. Scale bar is 25 μm.

Immunohistochemical staining for TNF-α revealed TNF-α positive Mφ and bronchiolar epithelial cells present at Day 1, in the lungs of animals instilled with 1.0 mg/kg Ag NWs especially in regions of heavy particle deposition and/or inflammation (Figure 11). At Day 7 (Figure 12), TGF-β-positive cells were prevalent in the lung parenchyma. By Day 21, Ag NW were primarily enclosed in granulomas or surrounded by numerous Mφ in the TB-alveolar duct junction, but TGF-β-positive cells (Figure 13) were still present.

### Silver localization

**Figure 14:**
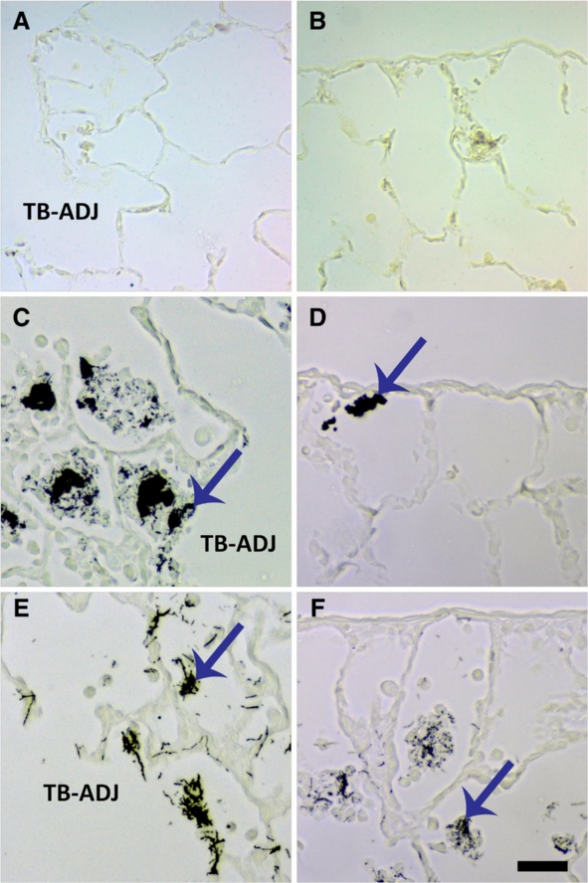
**Ag NW in lung tissues at Day 1.** Images are of autometallography -stained slides in the terminal bronchiole-alveolar duct junction [(TB-ADJ) **A**, **C**, **E**], and pleural regions (**B**, **D**, **F**). Tissues were obtained 1 day post instillation of sham control **(A-B)**, S-Ag NWs **(C-D)**, or L-Ag NWs **(E-F)** at 1.0 ml/kg. Ag NWs are indicated by blue arrows. Panels are Brightfield microscopy images. Scale bar is 25 μm.

**Figure 15:**
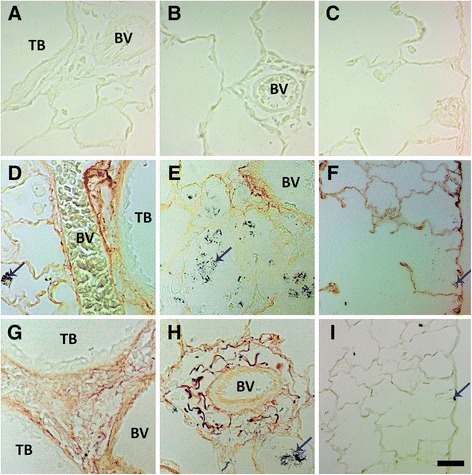
**Ag NWs and other silver species in lung tissues at Day 7.** Images are of autometallography -stained slides in the terminal bronchioles [(TB) left], perivascular (blood vessel) region [(BV) middle], and pleura (right). Tissues were obtained 7 days post instillation of sham control **(A-C)**, S-Ag NWs **(D-F)**, or L-Ag NWs **(G-I)** at 1.0 ml/kg. Ag NWs are indicated by blue arrows. Panels are Brightfield microscopy images. Scale bar is 25 μm.

**Figure 16:**
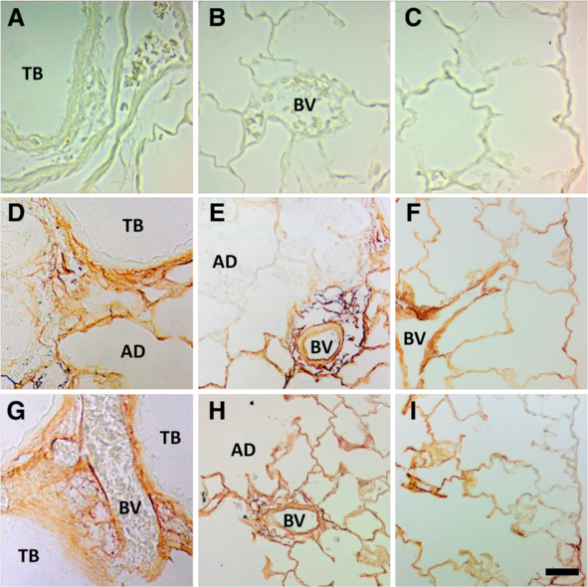
**Silver is still present in the lungs at Day 21.** Images are of autometallography -stained slides in the terminal bronchioles [(TB) left], perivascular (blood vessel) region [(BV) middle], and pleura (right). Tissues were obtained 21 days post instillation of sham control **(A-C)**, S-Ag NWs **(D-F)**, or L-Ag NWs **(G-I)** at 1.0 ml/kg. Panels are Brightfield microscopy images. AD = alveolar duct. Scale bar is 25 μm.

Autometallography showed that silver was still in the Ag NW particulate form at Day 1 and evident as fibrous and/or punctate black accumulations in the terminal bronchiole-alveolar duct junction (TB-ADJ) (Figure 14C & E), and pleural regions (Figure 14D & F). On Day 7, non-particulate silver was visibly radiating away from the black Ag NW agglomerates into adjacent tissues, and this ionic and/or protein-complexed silver left a characteristic halo of brown stain, which grew fainter with increased distance from the Ag NWs. Along with Ag NWs in the parenchyma, brown staining was observed on Day 7, in the sub-epithelial basement membrane of the airways (Figure 15D & G), extending through the parenchyma to perivascular and endothelial regions (Figure 15E & H), and/or out in the pleura (S-Ag NW-exposed animals only) (Figure 15F). Perivascular regions stained especially dark, which may indicate systemic translocation of silver. The pattern of staining observed on Day 7 continued to Day 21 (Figure 16); however, Ag NWs were not always visible in the vicinity of the brown staining, which, for L-Ag NW-exposed animals, finally did reach the pleura.

## Discussion

Fibrous HARNs are a class of engineered or naturally-occurring particles (e.g. Ag NWs and asbestos, respectively) that have a length to width ratio ≥3:1 [22], and at least one dimension (e.g. width) that is ≤100 nm [22]. Engineered HARNs are generating interest in the material sciences due to their unique physicochemical properties and wide-ranging applicability in fields like electronics and medicine. However, HARNs often exhibit relatively higher toxicity than their non-fibrous, particulate counterparts due to factors such as decreased clearance of the former versus the latter [22]. Results noted herein may be unique to Ag NW instillation in contrast to other HARNs. Instillation studies with titanium dioxide (TiO_2_) nanobelts [23,24] and carbon nanotubes [24-26] at similar dose ranges have produced mainly acute effects, and/or fewer inflammatory PMNs in BALF and/or lung tissues. These results contrast the foreign body reactions (Figures 5 and 9, Additional file 1: Figures S1, S6 & S7) and significant particle-associated inflammation (Additional file 1: Figure S5) noted through Day 21, and the presence of PMNs observed through Day 7 (Additional file 1: Figures S1D & F, and S7) in this study. However, at least one instillation study has reported evidence of foreign body reactions in tissues up to one month post exposure to single-walled carbon nanotubes [26], or recruitment of PMNs (eosinophils or neutrophils) up to four weeks post exposure to metal-containing NPs (but not their corresponding ions) [18]. Overall, findings suggest that more studies with Ag NWs are needed especially with occupationally relevant exposure conditions to discern whether these lasting and foreign body/granulomatous effects are due to bolus instillation doses. Instillation (rather than inhalation) was the chosen manner of exposure in this study because the delivery of a known Ag NW dose enabled the determination of dose responses. However, the delivered dose rate of a given dose as a bolus versus inhalation is different by orders of magnitude. Previous studies [25,27] showed that the immune inflammatory response is relatively less robust when the body is exposed to very small particles given at a low/steady rate in contrast to a large bolus. Therefore complementary inhalation studies with these Ag NWs should be performed.

Additionally, though human exposure to HARNs like Ag NWs are likely to be highest in occupational settings, where they are produced, packaged, and/or processed, the occurrence of low-level incidental exposures in the general public are still possible given the increasing use of Ag NW-containing consumer products (e.g. spray coatings, personal electronics, and clothing). It is therefore essential to fully understand the potential risks and factors associated with Ag NW exposure.

The “fibre paradigm” [22,28] distinguishes factors (dose, dimension, and durability) associated with pulmonary HARN toxicity. The dosage of HARNs to which an individual is exposed is essentially linked to his/her biological response. In this study, SD rats were instilled with 0, 0.1, 0.5, and 1.0 mg/kg Ag NWs. Given that significant inflammation (above control) was observed (in BALF and tissues) only with 0.5 and/or 1.0 mg/kg Ag NW instillations, these doses likely represent the lowest observed adverse effect level (LOAEL) for the biological responses measured herein.

Dimensions such as HARN length and diameter/width are key because thin (≤3 μm) [22], fibrous nanomaterials can traverse ciliated conducting airways and deposit deep in the lung parenchyma, and long nanomaterials (≥10 μm) [12,20] can inhibit clearance by alveolar Mφ. If the HARNs are also durable (biopersistant), not easily dissolved, or broken down into smaller pieces to allow clearance, accumulation of HARNs in the lungs may produce long-term effects (e.g. inflammation, fibrosis) and decrements in lung function [22].

Despite equal doses by mass, S-Ag NWs were shorter and narrower than L-Ag NWs, so higher numbers of S-Ag NWs were instilled [1.94, 9.68, and 19.37 (10^9^) S-Ag NWs/rat versus 0.05, 0.24, and 0.49 (10^9^) L-Ag NWs/rat]. This may explain the significantly greater cell numbers at Day 1 post IT of S-Ag NWs versus L-Ag NWs (Figure 2A, Additional file 1: Tables S1- S3). In contrasting responses produced by equivalent doses of S- and L-Ag NWs by mass, no significant differences were observed for BALF cell endpoints after Day 1, or for histopathology endpoints at any time. Indeed, the severity of responses to L-Ag NWs was often on par with those to S-Ag NWs.

Both S- and L-Ag NWs deposited in the deep lungs to produce significantly elevated numbers of particle-laden Mφ (Figure 3B & D, 0.5 and 1.0 mg/kg doses only) in contrast to the sham control, at Day 1. Total Mφ numbers in BALF did not increase, but signs of Mφ dysfunction (Figure 4) and/or death (Figure 5E-I, Additional file 1: Figure S1C-F) were observed at all time-points after Ag NW exposure. However, at Days 1 and 7, in L-Ag NW instilled animals, protein in BALF was significantly elevated above those exposed to S-Ag NWs (Figure 2D). Protein levels in the BALF are often correlated with the degree of cellular inflammation in the lungs [29]. Elevated BALF protein levels may indicate increased endothelial permeability associated with inflammation; Mφ signaling and/or necrosis due to frustrated phagocytosis; and/or damaged cells in the bronchiolar epithelium or alveoli. These early cellular responses post L-Ag NW instillation are certainly biologically plausible explanations for the findings observed herein. Only L-Ag NWs produced frustrated phagocytosis in Mφ from Days 1-21 (Figure 4, and Additional file 1: Figure S1E); frequent epithelial sloughing on Day 7 (Figure 7E); and repeated penetration/intercalation of the alveolar septal walls (Figure 14E & F) in Ag NW-instilled animals. Additionally, though histopathological scores suggest S-Ag NW instillation (0.5 or 1.0 mg/kg doses) may produce significant alveolar inflammation (Additional file 1: Table S5), only L-Ag NWs produced significant alveolar and bronchiolar inflammation in contrast to control (Table 3, Additional file 1: Table S6) at Days 1 and/or 7. Because the number of L-Ag NWs delivered was also at least one order of magnitude lower than that of S-Ag NWs, the greater potency of the former is especially evident. Despite this, both Ag NW types persisted to Day 21 (Figure 5F & I), and S- and L-Ag NW durability resulted in foreign body reactions (Figure 9) aimed at walling off the exogenous fibers. Because the S- and L-Ag NWs were also dissolving over time, it is possible that release of Ag^+^ (Figures 15 and 16) exacerbated the histopathology initiated by the bolus Ag NW instillation [15,17]. However, only after L-Ag NW exposure, on Day 21, were mesothelial cells and/or pleural Mφ observed protruding from the pleural lining into the pleural space (Additional file 1: Figure S6). Work by Murphy and colleagues [30] suggests that exposure to long nanomaterials produces frustrated phagocytosis in pleural macrophages resulting in amplification of pro-inflammatory cytokine responses by pleural mesothelial cells, and inflammation in the pleural cavity. For these reasons, L-Ag NWs may be more injurious than S-Ag NWs to lung Mφ and epithelial cells bearing the brunt of this injury.

Overall, histopathological responses herein were patchy, focal, and consistent with foreign body reactions. Foreign body responses are generally characterized by five main processes including 1) adsorption, 2) Mφ activation, 3) foreign-body giant cell (FBGC) formation, 4) fibroblast encapsulation, and 5) angiogenesis. When foreign materials enter the body, whether by purposeful (e.g. surgical implantation) or incidental (e.g. working in a dusty environment) means, the host’s biological response is essentially an attempt at removal and/or sequestration of these non-native bodies. Endogenous proteins e.g. fibrin or collagen aggregate around and adsorb to the foreign materials to create a biomolecular scaffolding. Macrophages attempt to phagocytose the foreign materials and produce numerous cytokines to stimulate other cells to aid in the healing process, which may involve inflammation and/or tissue remodeling mediated by cytokines like TNF-α, and TGF-β, respectively. Consequently, by Day 21, though Ag NWs were primarily contained in tissue granulomas or Langhans cells, fibrotic responses (Figure 9D & F), and TGF-beta expression (Figure 13D-I) was still strong. Results suggest that silver biopersistance (in the form of NWs and/or Ag^+^) may be affecting histopathology at these later time-points.

The findings of frustrated phagocytosis; cell death; significant increases in total BALF cells, protein, PMNs; and/or Ag NW-positive cells without a concomitant increase in Mφ suggest that S- and L-Ag NWs are at least acutely cytotoxic. Because Ag NWs are retained up to 21 days post instillation, Ag NW-associated inflammation and remodeling past Day 21 would be expected given the presence of Ag NWs and other silver species (likely ions and/or protein-complexed silver) as well as TGF-beta positive cells.

Variability in the responses produced by S- versus L-Ag NWs cannot assuredly be attributed in this study to differences in wire length alone. Rather, it is more likely that a combination of factors (e.g. particle length, dissolution rate, number) influenced differential responses. Our observations suggest that S- and L-Ag NW may be shedding Ag^+^ at different rates (Figure 15, Additional file 1: Figure S2), which is consistent with previous research [15,17]. Smaller (20 nm) PVP-coated Ag NPs produced more cellular toxicity and oxidative stress than larger (110 nm) Ag NPs because of a faster Ag dissolution rate and higher bioavailability in the former versus the latter. Thus, faster dissolution of S-versus L-Ag NWs at Day 1 post instillation may have also contributed to the relatively higher numbers of PMNs observed in the former versus the latter (Figure 2, Additional file 1: Tables S1 & S2). Abiotic silver dissolution studies performed on aliquots of the same Ag NWs used herein, in simulated freshwater [11] and cell culture media (manuscript in progress by Tagmount et al.)^a^, revealed greater Ag^+^ release within 48 hrs for S-Ag NWs versus L-Ag NWs. In addition, unidentified molecular components of Dulbecco's Modified Eagle Medium (DMEM), a protein-rich mammalian cell culture medium, enhanced the rate of oxidative dissolution and aggregation of Ag NWs^1^ (Additional file 1: Figure S8). However, abiotic Ag^+^ release rates are not always predictive of behavior *in vitro* because interactions between nanomaterials and components of biological fluids can affect stability in numerous ways. Scanlan and colleagues [11] found that Ag^+^ release in media could not account for observed Ag NW toxicity. For example, translocation of PVP-coated Ag NWs into Daphnid hemolymph led to marked morphological changes consistent with coating loss that was never observed in abiotic trials [11].

To more closely model occupational exposures, inhalation experiments should be performed with Ag NWs. Future studies contrasting Ag NWs of different lengths should consider using a particle number dose metric. Differences due to Ag NW size may affect dose by number, and/or silver dissolution (Ag^+^ release) rates. A simple, complementary durability study could be completed using gravimetric analysis of Ag NWs after incubation (at various time-points) in a simulated biological fluid (e.g. Gambles solution) [31]. This would approximate the contribution of Ag NW dissolution to fiber length and resulting biological responses. Quantification of different Ag species (in the lungs, lung-associated lymph nodes, and extra-pulmonary tissues), and biological responses (e.g. cyto-/chemo-kine release, cell dysfunction/death) post exposure will be most helpful in understanding how fate and clearance of silver correlate to effects *in vivo*. Quantification of Ag NWs and other silver species in tissues post Ag NW exposure may be achieved by X-ray microprobe analysis [32], atomic absorption spectrometry (AAS) [17], or single-particle inductively coupled plasma mass spectrometry (SP-ICP-MS) [33]. Pairing these analyses with measures of Mφ phagocytosis, chemotaxis, and/or activation of the NLRP3 inflammasome, which influences inflammation and apoptosis, would also be beneficial in deducing the pulmonary toxic potential of Ag NWs.

Future studies would also benefit from quantification of cytokines associated with foreign body inflammation (e.g., TNF-alpha, IL-1, monocyte chemoattractant protein-1); cell death and/or remodeling (e.g., TGF-beta); chemokines mediating neutrophil recruitment (e.g., LPS-induced CXC chemokine and monocyte chemoattractant protein-1); differentiation of apoptotic versus necrotic cells (e.g., Annexin V and Ethidium homodimer assays, respectively); and visual localization of dead/dying cells within the lungs (e.g., ethidium and confocal imaging). Analysis of the parietal pleura (in addition to the visceral pleura) should be completed as well. Because it can be difficult to maintain the integrity of the parietal pleura when extracting the lungs, histological analysis of the diaphragm [34] (a parietal pleural tissue surrogate) may be done. This, in combination with analysis of fluid and/or cells from the pleural cavity, would further elucidate the pleural effects of Ag NW exposure *in vivo*.

Overall, this study shows that Ag NWs produce 1) dose-dependent inflammation indicative of foreign body responses; 2) different inflammatory responses depending upon Ag NW length and/or dissolution rates; and 3) signs of lung remodeling at Days 7 and 21. Ag NWs persisted in the lungs through Day 21 and likely shed Ag ions, which have been shown previously to produce inflammatory responses apart from the parent Ag nanoparticles [15]. These factors suggest that Ag NWs may produce exposure-related health risks to humans especially at the occupational level where contact with Ag NWs can be higher. Given the increasing demand for products made with Ag NWs, further research must be done to ensure human health and safety as nanotechnology flourishes.

## Methods

### Physicochemical characterization of Ag nanowires

Aqueous suspensions of L-Ag NW and S-Ag NW were obtained from nanoComposix, Inc. (San Diego, CA) and stored under dark, anaerobic conditions at Lawrence Berkeley National Laboratory (LBNL). Aliquots of stock Ag NW were shipped in airtight 2.5-mL vials to UC-Davis for instillation studies. Both forms were synthesized using a heated ethylene glycol method and coated with 40 kD polyvinylpyrrolidone (PVP). L-Ag NWs (lot RKB4007) and S-Ag NWs (lot RKB3144) were imaged (Additional file 1: Figure S9A, B, & D) by transmission electron microscopy (TEM; S-Ag NWs only), and/or, scanning electron microscopy (SEM) finding good agreement with manufacturer specifications. SEM analysis of unused stock suspension returned to LBNL after instillation experiments showed no changes in NW morphology or aggregation state (Additional file 1: Figure S9E).

Total silver concentration of each suspension (Table 1) was measured in triplicate using ICP-MS after digesting 100-μL Ag NW aliquots in concentrated nitric acid. Quantification of dissolved silver (Ag^+^) in the stock (Table 1) was performed by filtering the suspension in 0.02-μm syringe filters and measuring silver in the filtrate. Studies of Ag^+^ release into aerobic water and other solutions over 24 hours have been described by Scanlan and colleagues [11]. At a total silver concentration of 110 μg/L, no silver release was detectable for L-Ag NW, while ~2 μg/L was detected for S-Ag NWs.

The dispersion state of the Ag NWs following dilution in ultrapure water was determined using dynamic light scattering (DLS; S-Ag only), and dark-field optical microscopy or SEM (S- and L-Ag NWs) by collecting onto cleaned silicon wafer substrates and imaging Ag NWs that settled from solution^a^. The observations established that both sizes of PVP-coated Ag NWs remained fully suspended in water prior to instillation.

### Animal protocol

Outbred male Sprague-Dawley rats (Harlan Laboratories, Inc., Hayward, CA), 11 weeks of age, were used for all experiments based on their high tolerance to intratracheal instillation procedures. Animals were kept at conditions previously described [23] in accordance with UC Davis Institutional Animal Care and Use Committee (IACUC). Prior to exposure, animals were randomly assigned to treatment groups. Animal weights were recorded throughout the study to monitor overt signs of distress. Sentinel rats were maintained in the same room and tested to ensure experimental animals remained free of pathogens and/or parasites.

### Preparation of Ag NW suspensions

Ag NW stock suspensions were double-sealed in 2.5 mL centrifuge tubes stored in scintillation vials, in a desiccator, under aseptic conditions, at room temperature until needed. Prior to use, stock suspensions were inverted 30× to ensure adequate particle dispersal. Direct dilutions of the stock Ag NW were done with nanopure H_2_0 to make lower concentration suspensions (0.1, 0.5 and 1.0 mg/ml) of the Ag NWs in 14 ml Falcon™ centrifuge tubes. Nanopure H_2_0 alone served as the sham control instillate. Dilutions were inverted 30× and loaded into 1cc Monoject® syringes fitted with 1.5 inch, 22 gauge, blunt-tipped Monoject® needles directly before instillation. Suspension preparation, syringe loading, and instillation were coordinated to ensure the highest degree of particle dispersion.

### Intratracheal instillation

Animals were instilled with Ag NW suspensions at 0, 0.1, 0.5 or 1.0 ml/kg bodyweight using procedures previously described [23,35]. Animals weighed approximately 0.35 kg, so the Ag NW doses tested herein (0.1, 0.5, and 1.0 mg/kg) corresponded to approximately 35, 175, and 350 μg/rat using a particle mass dose metric. Corresponding doses in particle numbers are presented in Table 2. For each Ag NW type, a sample size of 63 rats was used. There were six animals per dose (including controls) and 24 rats per time-point (1, 7, and 21 days post-exposure).

Schinwald et al. conducted one of the few studies to test Ag NWs in vivo [9] using C57BL/6 mice that were exposed to Ag NWs at a dose of 50 μg/mouse, and pulmonary inflammation resulted. The surface area of mouse alveolar epithelium (MAE) is 0.05 m^2^ [36]; thus, a mouse exposed to 50 μg via pharyngeal aspiration would have approximately 1000 μg Ag NWs/m^2^ MAE. The surface area of rat alveolar epithelium (RAE) is 0.4 m^2^ [36]. Doses tested herein, 0, 35, 175, and 350 μg/rat correspond to approximately 87.5, 437.5, and 875 μg Ag NWs/m^2^ RAE, respectively, and were chosen to confirm whether inflammatory effects could be produced at these lower doses.

At present, existing silver exposure limits are focused on the prevention of argyria, a non-toxic, cosmetic condition notable by a characteristic bluing of the skin. There are no known exposure limits for or occupational exposure studies measuring Ag NWs specifically. However, the American Conference of Governmental Industrial Hygienists (ACGIH) [37] set 8-hr time-weighted threshold limit values (TLVs) of 0.1 mg/m^3^ and 0.01 mg/m^3^ for metallic silver dust/fumes and soluble silver salts, respectively. Conservatively, the Occupational Safety and Health Administration (OSHA) [37], and the European Commission [38] set enforceable 8-hr time-weighted exposure limits at 0.01 mg/m^3^. Previous Ag NP sampling in an occupational area yielded a maximal time-weighted average of approximately 0.289 mg/m^3^ and an aerodynamic diameter range of 0.015-710.5 nm. Given:

A. 289 μg/m3 = experimentally measured concentration of Ag NP in the air [39]
B. 0.015-710.5 nm = aerodynamic diameter range of aerosolized Ag NPs [39]
C. 21.67 L/min = minute ventilation for a reference worker [40]
D. 10% = fraction of Ag NPs deposited into the human alveoli when breathing 1.0 μm particles [41]
E. 102 m^2^ = surface area of human alveolar epithelium (HAE) [36]

the approximate human occupational exposure after one month would be ~61.19 μg Ag NPs/m^2^ HAE. Therefore, the doses tested herein represent a worst-case-scenario.

### Collection and analysis of BALF and lung tissue samples

Animals were weighed and euthanized with an intraperitoneal injection of Beuthanasia-D (65 mg/kg) at 1, 7, or 21 days post Ag NW exposure. Broncho-alveolar lavage (BAL) was then performed. Briefly, the trachea was cannulated with a 16-gauge cannula. The left lung was clamped, and the right lung was lavaged 3× with a 9 mL aliquot of 0.9% sterile saline using a single 12 mL Monoject® syringe. Lavage fluid was collected in a 15 mL Falcon™ tube. Tubes were centrifuged at 4°C and 2000 rpm for 10 minutes (Beckman Coulter, Inc.). BAL supernatant from the 15 mL Falcon™ tube was collected for same-day protein analyses, which was performed using a kit from Sigma-Aldrich (St. Louis, MO). Cells from the 15 mL Falcon™ tube were resuspended in ~2 mL 0.9% sterile saline for determination of total cell numbers and cell viability with a hemocytometer and Trypan Blue exclusion dye, respectively. Autometallography was also done to identify silver particles in BAL Mφ. Cell differential and autometallography analyses were completed using Brightfield microscopy by counting a minimum of 500 cells from a 100 ml cytospin slide. Cell differential slides were stained with Diff Qwik® (Dade Behring Inc., Newark, DE); autometallography slides were stained using a silver enhancement kit for light and electron microscopy (Ted Pella, Redding CA) and a previously described method [15]. Blind analysis was then completed by two separate observers and compared to negate observer bias.

In addition to BALF, histological samples were collected from each animal and processed according to previously described methods [23]. Briefly, the left lung was fixed, micro-dissected, sliced, dehydrated and sectioned to 5 μm. Sections were then placed on slides for staining and cover-slipping to allow observations using Brightfield microscopy.

Hematoxylin (Harris Hematoxylin, American MasterTech, Lodi, CA) and eosin (Eosin Y Stain, American MasterTech, Lodi, CA) (H & E) staining was used to evaluate cellular changes and inflammation for all regions of the lungs with special emphasis on the examination of bronchiolar (terminal bronchioles, bronchoalveolar duct junctions), perivascular, alveolar, and pleural regions. Blind analysis was done by three individuals using a semi-quantitative, ordinal scoring system described previously [42]. The method was used to distinguish the degree of alveolar, bronchiolar, perivascular, particle-associated, and pleural inflammation in the H & E-stained tissue sections. In brief, scores ranging from 0-3, representing no, mild, moderate, and marked inflammation, respectively, were used. Severity was assessed by noting the most advanced grade present for each of the defined pathologies (alveolitis, bronchiolitis, etc.) within the specific sample irrespective of its horizontal extent. Extent was defined as the horizontal distribution of the pathology, where a score of 0, 1, 2, or 3 meant none of the lung was involved, ≤ 1/3 involvement, 1/2 involvement, or ≥ 2/3 involvement, respectively. Inflammatory extent was also characterized by descriptors “patchy” versus “continuous” and “focal” versus “diffuse”. However, the overall score was defined as a combined assessment of severity and extent (overall score = severity x extent). Because this method can be somewhat subjective, analysts underwent initial calibration training beforehand wherein each of the five specific histopathology categories were defined (Additional file 1: Table S4 and Figures S3-S4). Results from the three scorers were analyzed for agreement. Scores were analyzed separately for each of the five inflammation categories.

CEM (American MasterTech, Lodi, CA) stain was used to demonstrate eosinophilia in lung tissue sections. CEM selectively stains eosinophilic granules a bright red/pink color and has the best specificity for distinguishing eosinophils from neutrophils compared to other histochemical stains [43]. Immunohistochemistry (IHC) assays for presence of Tumor Necrosis Factor-Alpha (TNF-α) (American MasterTech) and Transforming Growth Factor-Beta (TGF-β) (American MasterTech) were performed to pinpoint acute inflammatory and later fibrotic changes in the lungs, respectively. Autometallography was done to determine silver distribution.

### Statistical analyses

All BALF data sets (total cells, cell differentials, and total protein) were analyzed using JMP statistical software (Cary, NC). Outliers were identified via box plots. Data were then analyzed for deviations from the assumptions of Analysis of Variance (ANOVA) using Levene and Bartlett tests to confirm homoscedasticity and Shapiro-Wilk tests on model residuals to confirm normality. When the assumptions were met, ANOVA at *p* ≤ 0.05 (confidence interval [CI] 95%) was used to consider the main effects of and interactions between the independent variables (dose, time/day, and/or particle length/type). *Post hoc* Tukey HSD tests were also performed to determine specific significant differences. Graphical data are presented as mean ± standard error of the mean (SEM). Descriptive statistics are presented in text and/or tables. Values shown in tables were from Tukey HSD Multiple Comparisons. *P*-values were rounded up to 0.05, 0.01, or 0.001.

Agreement among the three histopathology raters was assessed using Kappa measurements of precision. Though pathology scores were ordinal, these data were treated as continuous after confirming ANOVA assumptions were met. ANOVA instead of categorical analyses was run on histopathology scores to 1) enable tests of interactions between independent variables; and 2) control for Type I and II errors, which can be incurred by multiple separate non-parametric analyses.

## Endnote

^a^Tagmount, A., Gilbert, B., Loguinov, A.V., and Vulpe, C.D. Silver nanowire characterization and toxicity. (in progress).

## Additional file

Additional file 1: Figure S1. Ag NW instillation produces an influx of PMNs and phenotypic changes in macrophages. **Figure S2.** Variety of staining suggests multiple silver species in cells. **Figure S3.** Illustrated Histopathology Scoring Reference: Part I. **Figure S4.** Illustrated Histopathology Scoring Reference: Part II. **Figure S5.** Ag NWs produce significant particle-associated inflammation at all time-points. **Figure S6.** L-Ag NW-induced pleural inflammation at Day 21. **Figure S7.** At Day 7, inflammatory PMNs were still present in Ag NW-exposed tissues. **Figure S8.** After one week in protein-rich media, Ag NW agglomerates (A) are detectable, but individual NWs (B) are still present. **Figure S9.** Transmission electron microscopy (TEM) and scanning electron microscopy (SEM) characterization of PVP-coated Ag NWs after receipt from nanoComposix, Inc. and following different periods in storage. **Table S2.** Significant Post Hoc Tukey HSD Comparisons of Interactions: BALF Total PMNs. **Table S3.** Significant Post Hoc Tukey HSD Multiple Comparisons: BALF Total Cells. **Table S4.** Histopathology Scoring Rubric. **Table S5.** Significant Post Hoc Tukey HSD Comparisons: S-Ag NW Induced Histopathology. **Table S6.** Significant Post Hoc Tukey HSD Comparisons of Interactions (Dose * Day): L-Ag NW Induced Histopathology.
