# Supplementary material for: Short versus long silver nanowires: a comparison of in vivo pulmonary effects post instillation
Source: Part Fibre Toxicol. 2014 Oct 8;11:52. doi: 10.1186/s12989-014-0052-6 (PMC4198797; doi:10.1186/s12989-014-0052-6)
Supplement: Additional file 1: Figure S1. — Ag NW instillation produces an influx of PMNs and phenotypic changes in macrophages. Figure S2. Variety of staining suggests multiple silver species in cells. Figure S3. Illustrated Histopathology Scoring Reference: Part I. Figure S4. Illustrated Histopathology Scoring Reference: Part II. Figure S5. Ag NWs produce significant particle-associated inflammation at all time-points. Figure S6. L-Ag NW-induced pleural inflammation at Day 21. Figure S7. At Day 7, inflammatory PMNs were still present in Ag NW-exposed tissues. Figure S8. After one week in protein-rich media, Ag NW agglomerates (A) are detectable, but individual NWs (B) are still present. Figure S9. Transmission electron microscopy (TEM) and scanning electron microscopy (SEM) characterization of PVP-coated Ag NWs after receipt from nanoComposix, Inc. and following different periods in storage. Table S2. Significant Post Hoc Tukey HSD Comparisons of Interactions: BALF Total PMNs. Table S3. Significant Post Hoc Tukey HSD Multiple Comparisons: BALF Total Cells. Table S4. Histopathology Scoring Rubric. Table S5. Significant Post Hoc Tukey HSD Comparisons: S-Ag NW Induced Histopathology. Table S6. Significant Post Hoc Tukey HSD Comparisons of Interactions (Dose * Day): L-Ag NW Induced Histopathology. [file 12989_2014_52_MOESM1_ESM.pdf]

Supplemental Figures and Tables

Short versus long silver nanowires: a comparison of *in vivo* pulmonary effects post instillation

Rona M. Silva[1], Jingyi Xu[1, 2], Clare Saiki[1], Donald S. Anderson[1], Lisa M. Franzi[3], Chris D. Vulpe[4], Benjamin Gilbert[5], Laura S. Van Winkle[1], and Kent E. Pinkerton[1]*

[1]Center for Health and the Environment, University of California, Davis, CA 95616. Email: lusilva@ucdavis.edu, dlxujingyi@163.com, claresaiki@sbcglobal.net, dsanderson@ucdavis.edu, lsvanwinkle@ucdavis.edu

[2] Department of Endocrinology, Affiliated Zhongshan Hospital of Dalian University, Dalian, Liaoning, 116001. China. Email: dlxujingyi@163.com

[3]Department of Pulmonary Medicine, School of Medicine, University of California, Davis, CA 95616. Email: lisa.franzi@ucdmc.ucdavis.edu

[4] Department of Nutritional Science and Toxicology, University of California, Berkeley, CA, 94720. Email: vulpe@berkeley.edu

[5] Geochemistry Department, Berkeley Nanogeoscience Center, University of California, Berkeley, CA, 94720. Email: bgilbert@lbl.gov

*Correspondence: Dr. Kent E. Pinkerton, Tel: (530) 752-8334. Email: kepinkerton@ucdavis.edu

Supplemental Table S1. Significant Post Hoc Tukey HSD Comparisons of Main Effects: BALF Total PMNs

Factor of Comparison	Group I (A)	Group II (B)	Mean Difference (A-B)	Standard Error Difference (A-B)	*p* - value	*df*	Lower CL	Upper CL
**Main Effects: Total Neutrophils**								
Dose	0.5 mg/kg	Control	21.60	2.77	<0.0001	3	14.38	28.83
Dose	1.0 mg/kg	Control	11.01	2.77	<0.001	3	3.78	18.24
Day	Day 1	Day 7	21.50	2.41	<0.0001	2	15.78	27.23
Day	Day 1	Day 21	24.91	2.40	<0.0001	2	19.22	30.6
Length	S-Ag NW	L-Ag NW	5.24	1.97	<0.01	1	1.34	9.14
**Main Effects: Total Eosinophils**								
Dose	0.5 mg/kg	Control	1.82	0.61	<0.05	3	0.22	3.42
Dose	1.0 mg/kg	Control	3.44	0.61	<0.0001	3	1.842	5.05
Day	Day 1	Day 7	3.76	0.53	<0.0001	2	2.50	5.03
Day	Day 1	Day 21	3.94	0.53	<0.0001	2	2.68	5.20

ANOVA model of BALF total PMN data tested the main effects of particle length, Ag NW dose, and time post instillation (day). Values above were obtained from Tukey HSD Multiple Comparisons. *P* values were rounded up to 0.5, 0.01, or 0.001.

Results above show that Ag NW dose, length*, and the amount of time post instillation (day) were significant factors with respect to inflammatory polymorphonuclear cells [(PMNs) neutrophils and/or eosinophils] in BALF. S-Ag NW instillation resulted in significantly more neutrophils than L-Ag NW (M = 12.56 and M = 7.33, respectively), and the total number of PMNs was statistically higher at Day 1 than at Days 7 and 21.

*Significant effects due to length alone (Supplemental Table S1 above), and due to the interaction of length and day (Supplemental Table S2) were noted. However, testing the interaction of dose, Ag NW length, and day altogether (Figure 1, main text) did not show significant differences between S- and L-Ag NW-induced BALF neutrophilia. Thus, the statistical model suggests that length may have a marginal effect on the number of BALF neutrophils recovered post instillation when using particle mass (mg/kg bodyweight) dose metrics.

Supplemental Table S2. Significant Post Hoc Tukey HSD Comparisons of Interactions: BALF Total PMNs

Factors of Comparison	Group I (A)	Group II (B)	Mean Difference (A-B)	Standard Error Difference (A-B)	*p* - value	*df*	Lower CL	Upper CL
**Interactions: Total Neutrophils**								
Day*Dose	Day 1, 0.5 mg/kg	Day 7, 0.5 mg/kg	24.06	4.88	<0.001	6	7.78	40.34
Day*Dose	Day 1, 0.5 mg/kg	Day 21, 0.5 mg/kg	30.00	4.76	<0.0001	6	14.11	45.89
Day*Dose	Day 1, 1.0 mg/kg	Day 7, 1.0 mg/kg	51.73	4.76	<0.0001	6	35.85	67.62
Day*Dose	Day 1, 1.0 mg/kg	Day 21, 1.0 mg/kg	59.29	4.88	<0.0001	6	43.01	75.57
Length*Day	S-Ag NW, Day 1	L-Ag NW, Day 1	12.00	3.37	<0.01	2	2.24	21.76
Length*Day	S-Ag NW, Day 1	S-Ag NW, Day 7	25.90	3.41	<0.0001	2	16.02	35.79
Length*Day	S-Ag NW, Day 1	S-Ag NW, Day 21	30.65	3.37	<0.0001	2	20.89	40.41
Length*Day	L-Ag NW, Day 1	L-Ag NW, Day 7	17.10	3.41	<0.0001	2	7.22	26.98
Length*Day	L-Ag NW, Day 1	L-Ag NW, Day 21	19.16	3.41	<0.0001	2	9.28	29.04
**Interactions: Total Eosinophils**								
Day*Dose	Day 1, 0.5 mg/kg	Day 7, 0.5 mg/kg	5.22	1.08	<0.001	6	1.61	8.83
Day*Dose	Day 1, 0.5 mg/kg	Day 21, 0.5 mg/kg	5.33	1.05	<0.0001	6	1.82	8.85
Day*Dose	Day 1, 1.0 mg/kg	Day 7, 1.0 mg/kg	9.17	1.05	<0.0001	6	5.65	12.69
Day*Dose	Day 1, 1.0 mg/kg	Day 21, 1.0 mg/kg	9.75	1.08	<0.0001	6	6.15	13.36

ANOVA model of BALF total PMN data tested interactions between particle length, Ag NW dose, and time post instillation (day). Values above were obtained from Tukey HSD Multiple Comparisons. *P* values were rounded up to 0.5, 0.01, or 0.001.

There was a strong effect due to the interaction between Ag NW dose and time post instillation (day) with the 0.5 and 1.0 mg/kg doses producing significantly different degrees of PMN influx at Day 1, than at Days 7, and 21. The interaction between particle length and time was also significant as neutrophilia was higher at Day 1 than Day 7 for both S- and L-Ag NW (M = 5.51 and M = 2.31, respectively) and Day 21 (M = 0.76 and M = 0.25, respectively).

Supplemental Table S3. Significant Post Hoc Tukey HSD Multiple Comparisons: BALF Total Cells

Factor(s) of Comparison	Group I (A)	Group II (B)	Mean Difference (A-B)	Standard Error Difference (A – B)	*p* - value	*df*	Lower CL	Upper CL
**Main Effects**								
Length	S-Ag NW	L-Ag NW	8.80	3.35	<0.01	1	2.16	15.44
Dose	1.0 mg/kg	Control	25.90	4.72	< 0.0001	3	13.60	38.20
Day	Day 1	Day 7	26.24	4.10	< 0.0001	2	16.50	35.99
Day	Day 1	Day 21	29.55	4.08	< 0.0001	2	19.86	39.23
**Interactions**								
Length*Day	S-Ag NW, Day 1	L-Ag NW, Day 1	20.47	5.73	<0.01	2	3.86	37.09
Length*Day	S-Ag NW, Day 1	S-Ag NW, Day 7	31.41	5.80	< 0.0001	2	14.60	48.24
Length*Day	S-Ag NW, Day 1	S-Ag NW, Day 21	41.88	5.73	< 0.0001	2	25.26	58.49
Length*Day	L-Ag NW, Day 1	L-Ag NW, Day 7	21.06	5.80	<0.01	2	4.24	37.88
Length*Day	L-Ag NW, Day 1	L-Ag NW, Day 21	17.21	5.80	<0.05	2	0.40	34.03
Day*Dose	Day 1, 1.0 mg/kg	Day 7, 1.0 mg/kg	57.99	8.11	< 0.0001	6	30.95	85.04
Day*Dose	Day 1, 1.0 mg/kg	Day 21, 1.0 mg/kg	77.4	8.31	< 0.0001	6	49.69	105.2

ANOVA model of BALF total cell data tested the effects of and interactions between particle length, Ag NW dose, and time post instillation (day). Values above were obtained from Tukey HSD Multiple Comparisons. *P* values were rounded up to 0.5, 0.01, or 0.001.

In addition to the main effects mentioned in the main text, significant interactions between the factors, particle length and day [$F(2) = 4.60$, $p = 0.01$], and between day and dose [$F(6) = 9.54$, $p < 0.0001$] were also found in the ANOVA model. Instillation of S-Ag NWs ($M = 84.74$) produced significantly greater cells than L-Ag NWs at Day 1 ($M = 64.27$), and S-Ag NWs at Days 7 ($M = 53.32$) and 21 ($M = 42.86$). L-Ag NW exposure also produced more cells at Day 1 than Days 7 ($M = 43.20$) and 21 ($M = 47.05$). Further, instillation of 1.0 mg/kg Ag NWs specifically, produced significantly more cells at Day 1, than at Days 7 and 21.

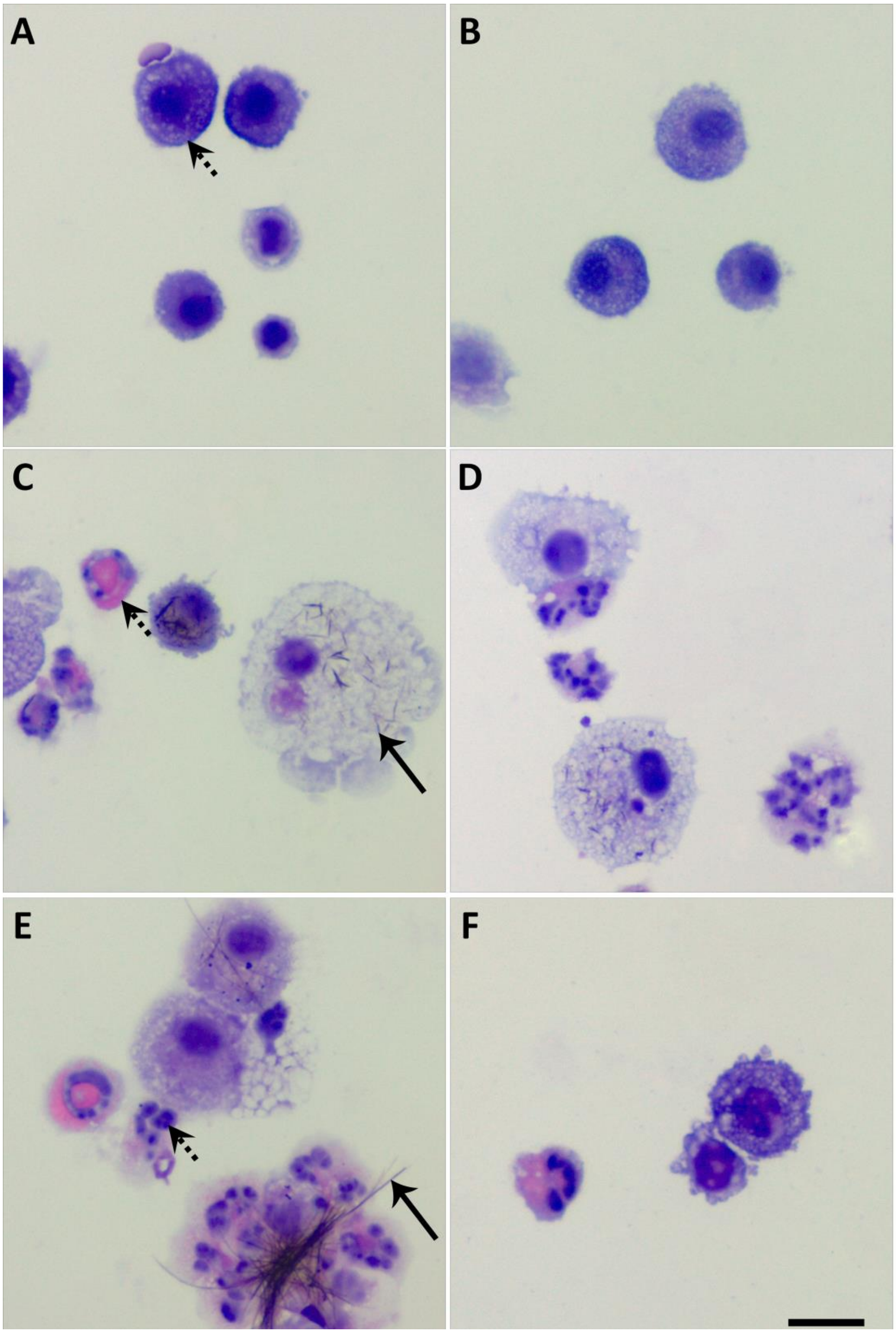

Supplemental **Figure S1. Ag NW instillation produces an influx of PMNs and phenotypic changes in macrophages.** Cells recovered from BALF at 1 day (left panels) and 7 days (right panels) post exposure to sham control (A & B), S-Ag NWs (C & D), or L-Ag NWs (E & F). All panels are Brightfield microscopy images of cells from rats given a single instillation of sham control or Ag NW suspension at 1.0 mL/kg. BAL cells were stained with Diff Qwik®. Different cell types are noted once by broken arrows for reference. Macrophages (A) have large cytoplasm and a single dark nucleus. Eosinophils (C) stain bright pink and have a dark bi-lobular (sometimes doughnut-shaped) nucleus. Neutrophils (E) stain light blue/purple and have a dark multi-lobular nucleus. Ag NWs are indicated by solid arrows. Scale bar is 25 µm.

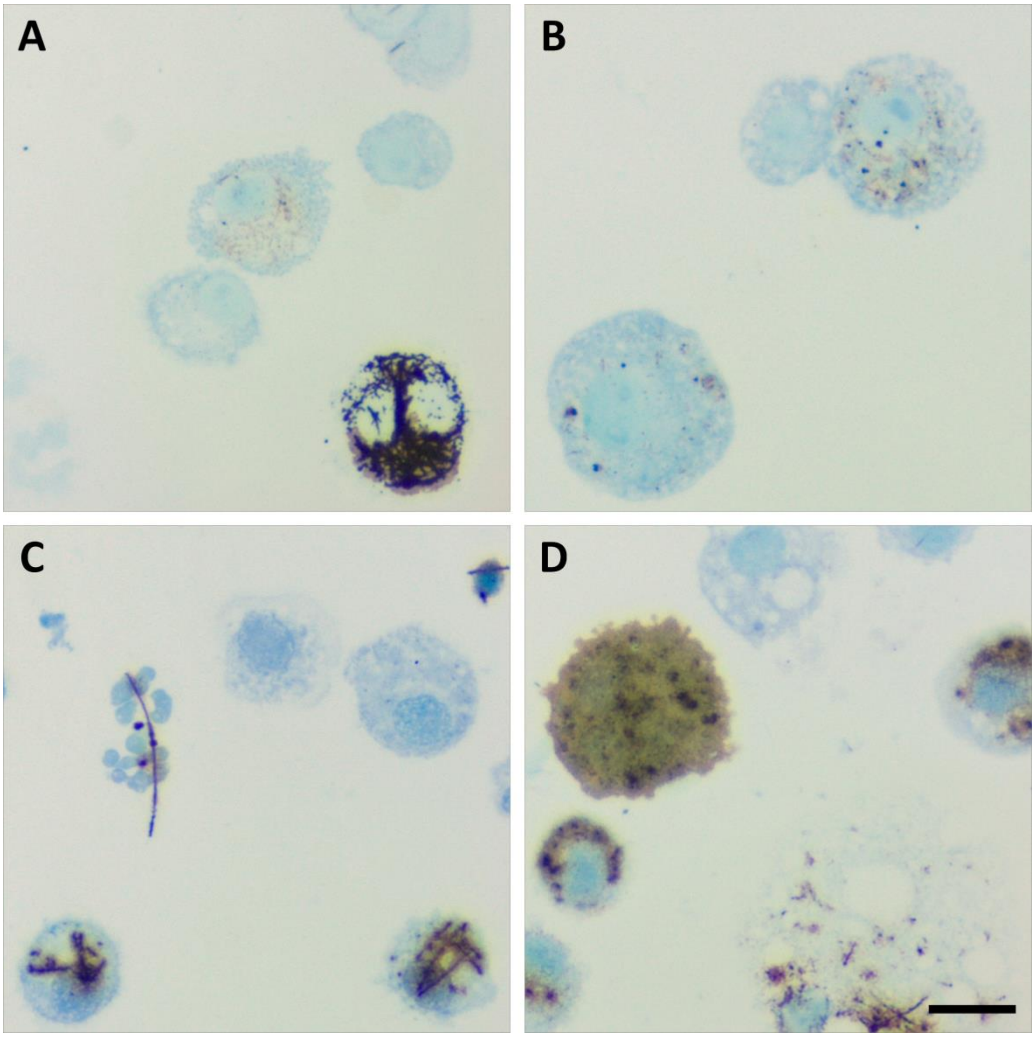

Supplemental Figure S2. Variety of staining suggests multiple silver species in cells.

Cells recovered from BALF post exposure post exposure to S-Ag NWs at Days 1 and 7(A & B, respectively), or L-Ag NWs at Days 1 and 21(C & D, respectively). All panels are Brightfield microscopy images of cells from rats given a single instillation of Ag NWs at 1.0 mg/kg. BAL cells were stained with autometallography and toluidine blue counter-stain. Scale bar is 25 µm.

Supplemental Table S4. Semi-Quantitative Histopathology Scoring Rubric

Semi-Quantitative Histopathology Scoring Rubric

Type of Pathology	Score			
	0	1	2	3
**Alveolitis**	Normal. Thin alveolar walls, with very few free macrophages in the lumen. No inflammatory cells.	Similar to 0 score with more free macrophages in the alveolar lumen. No PMNs.	Atypical cellularity in the walls and/or lumen of the alveoli with the majority of the alveolar spaces still clear of free cells. Over-represented cell types include macrophages, monocytes, and/or PMNs.	Thickened alveolar walls. Marked influx of mixed cells (phagocytes and/or PMNs) into the alveolar lumen forming large cellular agglomerates which occupy much of the airspace.
**Bronchiolitis**	Normal respiratory epithelium, 1 cell-layer thick. Normal smooth muscle and submucosal layers.	Mild influx of macrophages and/or monocytes to the airway submucosa, but no PMNs.	Slightly thickened airway due to moderate influx of PMNs and/or phagocytes into the submucosa. PMNs encompass <15% of influxing cells.	Marked influx of inflammatory cells into the submucosal layer causing pronounced thickening of the airway. A high percentage of PMNs may be present, but is not necessary.
**Perivascular Inflammation**	Normal vascular endothelium.	Mild influx of a few macrophages and/or monocytes to the region, but no PMNs. Nearly all of the connective tissue is still visible.	Moderate PMN and/or phagocyte infiltrates with much of the connective tissue still visible.	Marked mixed cellular infiltrates such that much of the connective tissue is obscured by influxing cells. A high percentage of PMNs may be present.
**Particle Agglomerates**	No particle agglomerates.	Obvious particle agglomerate with little/no increase in vicinal cellularity.	Obvious particle agglomerate with moderate increase in vicinal cellularity. Phagocyte and/or PMN influx. Small cellular aggregates possible.	Obvious particle agglomerate surrounded by large, focal cellular infiltrates.
**Pleural Inflammation**	Little/no cells at the pleura.	Slightly increased cellularity at the pleura. No PMNs.	Moderately increased cellularity with PMNs and/or phagocytes.	Severe influx of cells to the pleura. A high percentage of PMNs may be present along with foamy macrophages.

Supplemental Figure S3. Illustrated Histopathology Scoring Reference: Part I

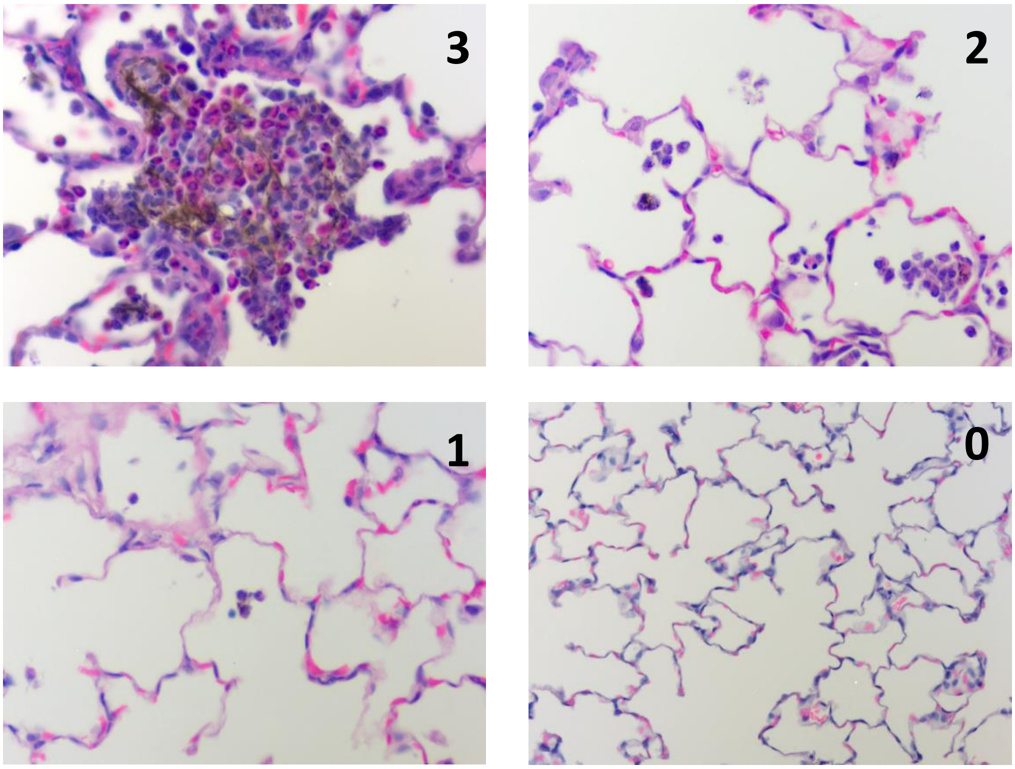

Alveolitis

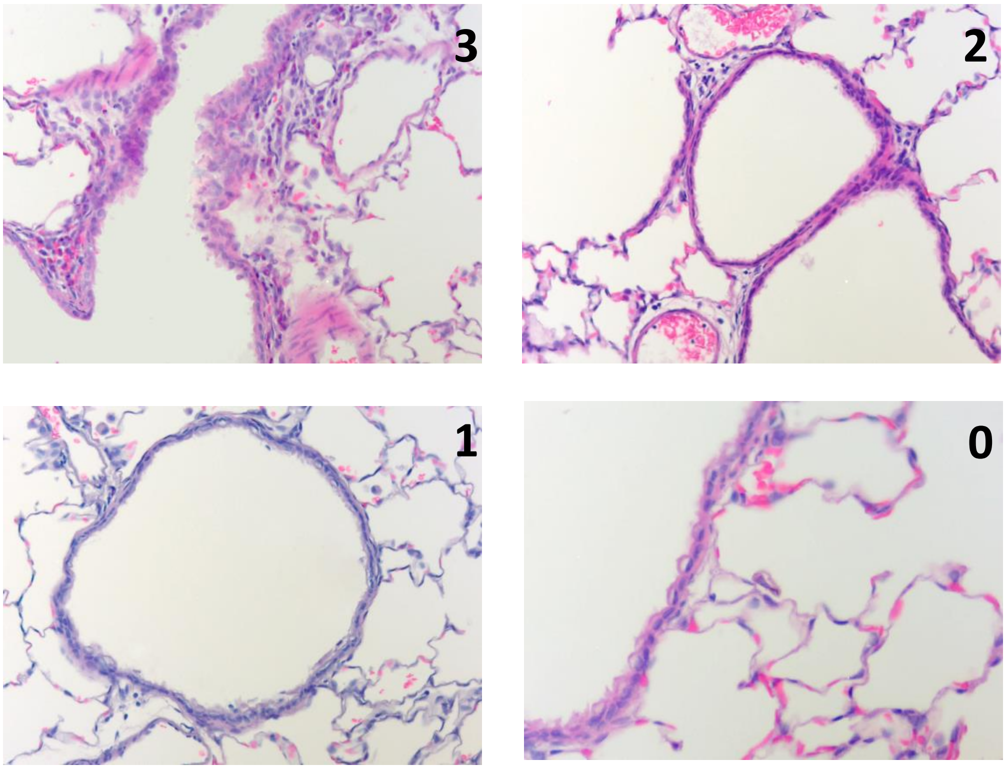

Bronchiolitis

Supplemental Figure S4. Illustrated Histopathology Scoring Reference: Part II

Perivascular Inflammation

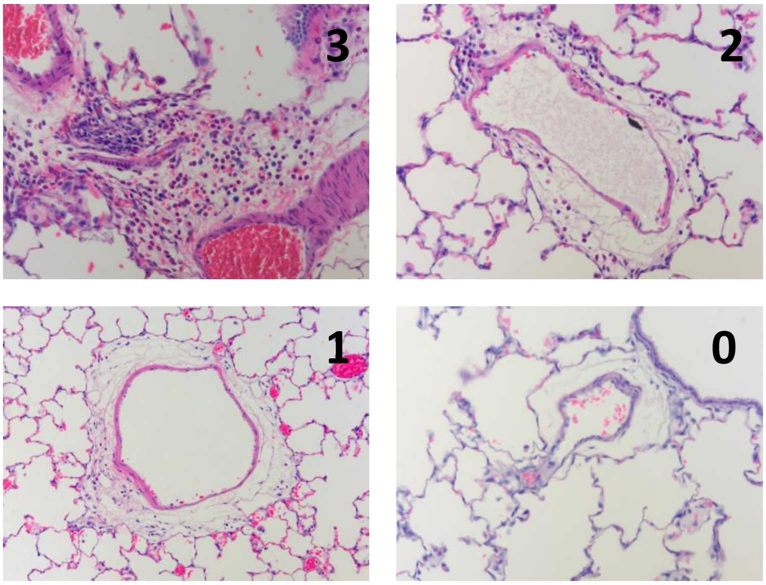

Particle-Associated Inflammation

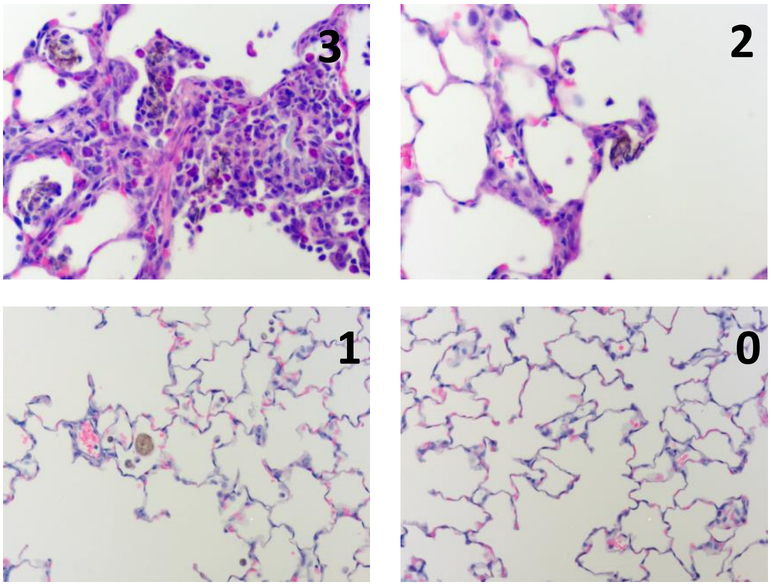

Pleural Inflammation

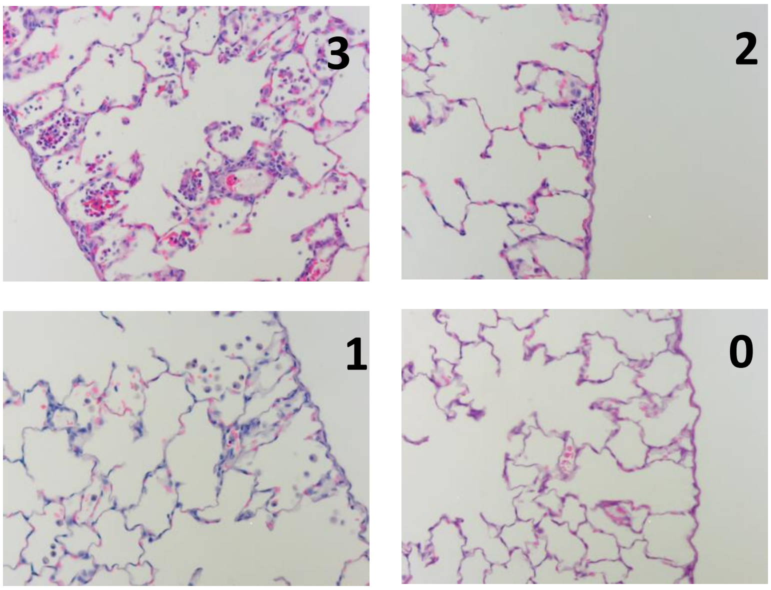

Supplemental Table S5. **Significant Post Hoc Tukey HSD Comparisons: S-Ag NW Induced Histopathology**

Type of Inflammation Compared	Group I (A)	Group II (B)	Mean Difference (A-B)	Standard Error Difference (A – B)	*p* - value	*df*	Lower CL	Upper CL
**Main Effect = Dose**								
Alveolar	0.5 mg/kg, M = 2.49	Control, M = 1.78	0.71	0.20	<0.01	3	0.20	1.22
Alveolar	1.0 mg/kg, M = 2.33	Control, M = 1.78	0.56	0.20	<0.05	3	0.03	1.08
**Main Effect = Day**								
Alveolar	Day 21, M = 2.51	Day 1, M = 1.95	0.56	0.16	<0.01	2	0.17	0.94
Alveolar	Day 21, M = 2.51	Day 7, M = 2.02	0.49	0.17	0.01	2	0.10	0.89
Bronchiolar	Day 21, M = 2.95	Day 1, M = 2.64	0.31	0.08	<0.001	2	0.11	0.51
Pleural	Day 21, M = 2.30	Day 1, M = 1.74	0.57	0.16	0.001	2	0.20	0.94
Pleural	Day 21, M = 2.30	Day 7, M = 1.79	0.51	0.16	<0.01	2	0.13	0.89
Perivascular	Day 21, M = 2.70	Day 7, M = 2.24	0.46	0.12	<0.001	2	0.17	0.75

ANOVA model of Histopathology scoring data tested the effects of and interactions between S-Ag NW dose, and time post instillation (day). Values above were obtained from Tukey HSD Multiple Comparisons. P values were rounded up to 0.5, 0.01, or 0.001. CON = "convincing" findings, $p < 0.0001$.

Results above show that Ag NW dose and the amount of time post instillation (day) were significant factors with respect to S-Ag NW-induced histopathologies scored using a semi-quantitative method. Testing the interaction of dose and day together did not show significant differences. (In other words, upon testing whether the doses were equally inflammatory at the various time-points, we found no significant differences.)

We conclude from this data that:

- Instillation of 0.5 or 1.0 mg/kg S-Ag NWs resulted in significantly more alveolar inflammation than controls.
- Alveolar, bronchiolar, pleural, and perivascular inflammation seemed to increase with time post S-Ag NW instillation.
- The factors (dose and day) have marginal effects on the histopathology observed post instillation of S-Ag NWs.

Supplemental Table S6. Significant Post Hoc Tukey HSD Comparisons of Interactions (Dose * Day): L-Ag NW Induced Histopathology

Type of Inflammation Compared	Group I (A)	Group II (B)	Mean Difference (A-B)	Standard Error Difference (A – B)	*p* - value	*df*	Lower CL	Upper CL
Alveolar	0.5 mg/kg, Day 1 *M* = 3.00	Control, Day 1 *M* =1.44	1.56	0.32	0.001	6	0.47	2.69
Alveolar	1.0 mg/kg, Day 1 *M* = 2.58	Control, Day 1 *M* =1.44	1.14	0.32	0.05	6	0.06	2.22
Alveolar	1.0 mg/kg, Day 7 *M* =3.00	Control, Day 7 *M* =1.67	1.33	0.32	0.01	6	0.25	2.42
Alveolar	1.0 mg/kg, Day 7 *M* = 3.00	0.1 mg/kg, Day 7 *M* =1.42	1.58	0.30	CON	6	0.58	2.59
Bronchiolar	0.5 mg/kg, Day 7 *M* = 3.00	Control, Day 7 *M* =2.44	0.56	0.15	0.05	6	0.05	1.06

ANOVA model of histopathology scoring data tested the effects of and interactions between L-Ag NW dose, and time post instillation (day). Values above were obtained from Tukey HSD Multiple Comparisons. *P* values were rounded up to 0.5, 0.01, or 0.001. CON = "convincing" findings, $p < 0.0001$.

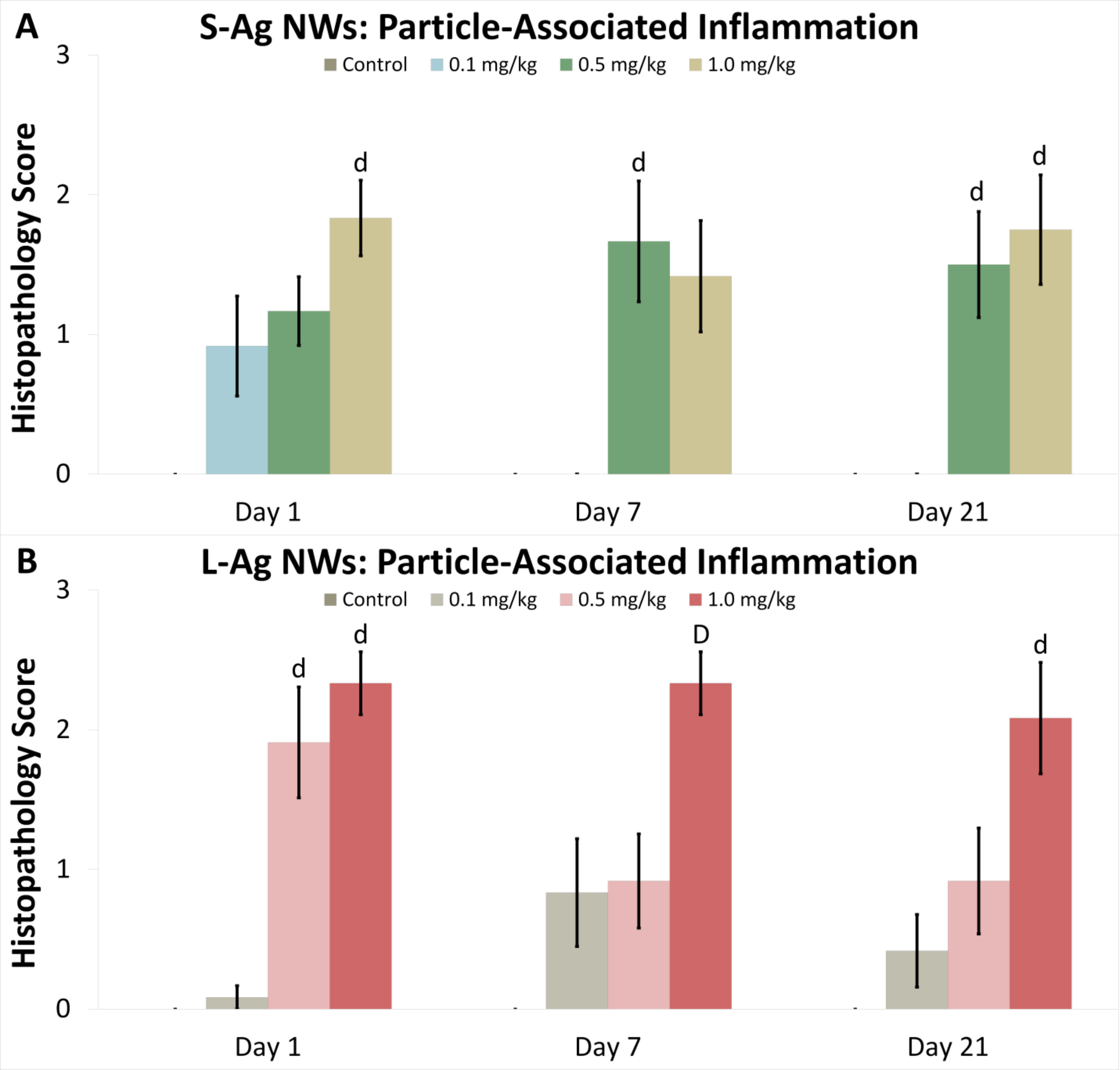

Supplemental Figure S5. Ag NWs produce significant particle-associated inflammation at all time-points. Graphs show semi-quantitative scoring for S- and L-Ag NWs (A and B, respectively). Results are from ANOVAs considering the interaction of factors, dose and time post instillation, at $p \leq 0.05$. "D" and "d" indicate significant differences between groups (in the same panel) given different doses of the same Ag NWs, but sacrificed on the same day. "D" corresponds specifically to differences between groups given 1.0 mg/kg Ag NWs versus any other dose; while, "d" indicates differences from the sham control and 0.01 mg/kg groups only.

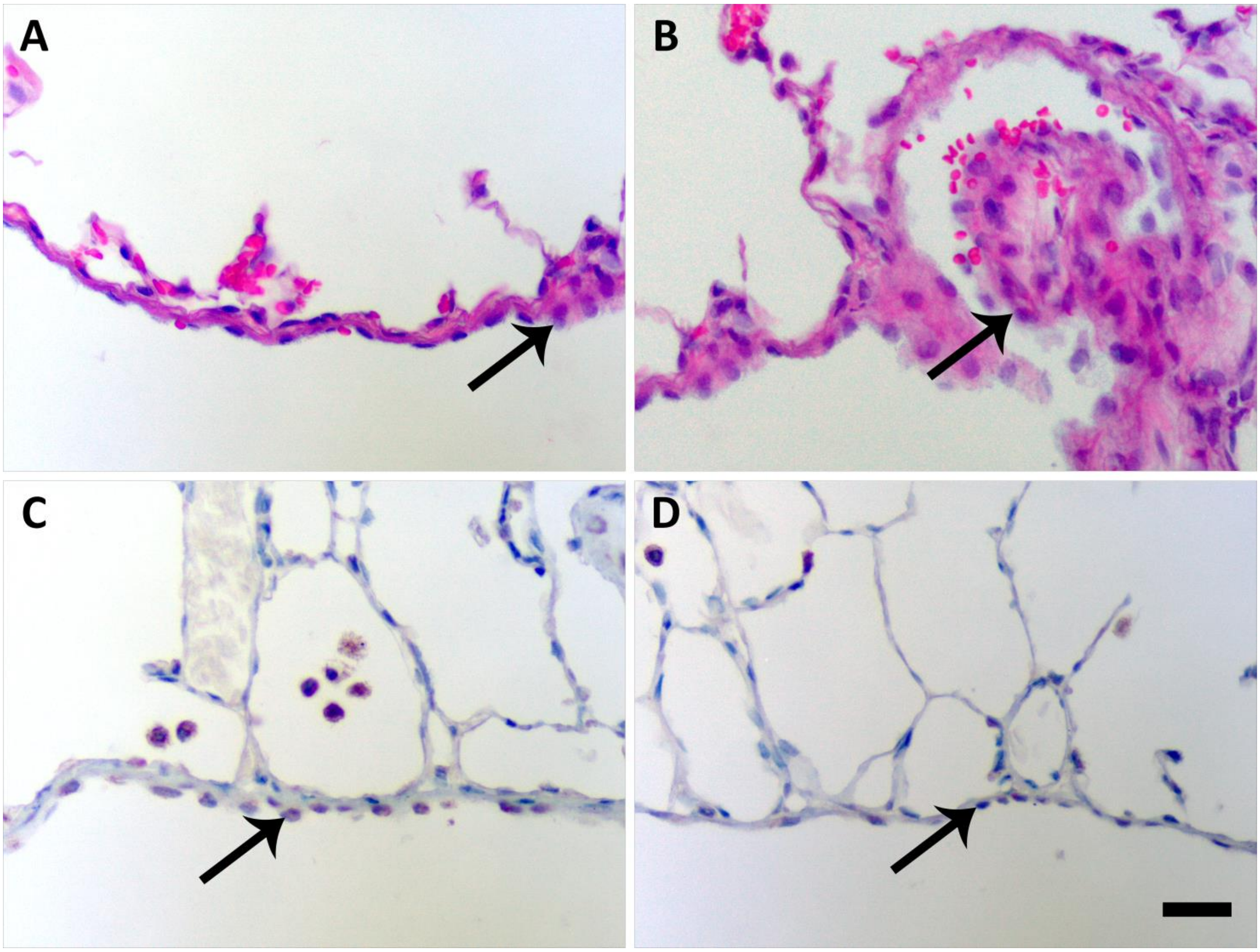

Supplemental Figure S6. L-Ag NW-induced plural inflammation at Day 21. Images are H & E- (A-B) or TNF-α-stained (C-D) tissue sections recovered at Day 21 post exposure to L-Ag NWs. Panels are Brightfield microscopy images of responses in tissues from rats instilled with a single dose of 1.0 mg/kg L-Ag NWs. Solid arrows indicate thickened pleural tissue [most severe case observed (A-B)] or cells protruding from the pleural lining (C-D). (Findings were not observed upon S-Ag NW instillation). Scale bar is 25 µm.

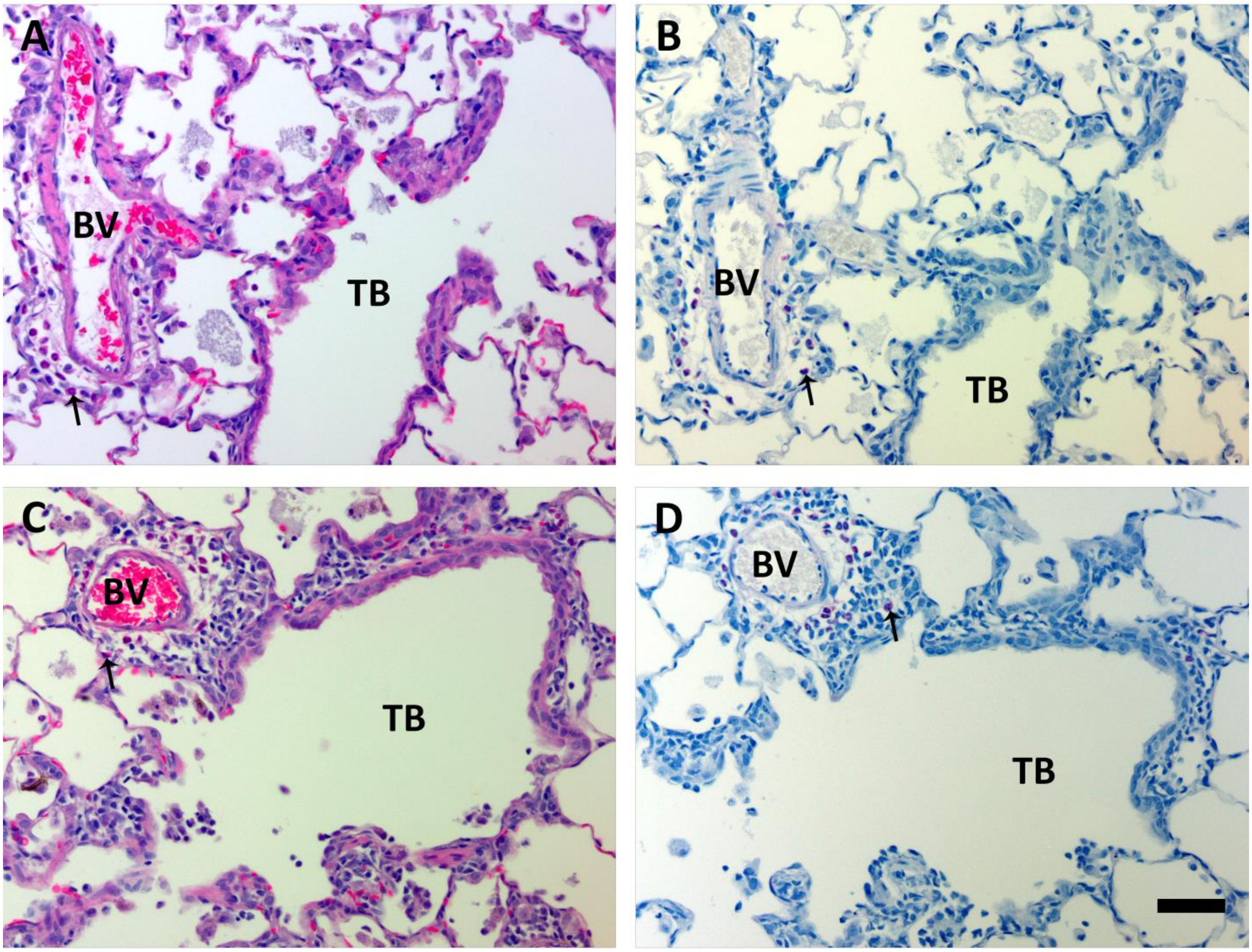

Supplemental Figure S7. At Day 7, inflammatory PMNs were still present in Ag NW-exposed tissues. Images are from serial tissue sections stained with H & E (left) or CEM (right) stains, and recovered at 7 days post instillation of S-Ag NWs (A-B), or L-Ag NWs (C-D). Panels are Brightfield microscopy images of tissues from rats instilled with a single 1.0 ml/kg dose of Ag NWs. Arrows indicate eosinophils, which are bright pink in H & E and CEM panels. BV = blood vessel, and TB-ADJ = terminal bronchiole-alveolar duct junction. Scale bar is 50 µm.

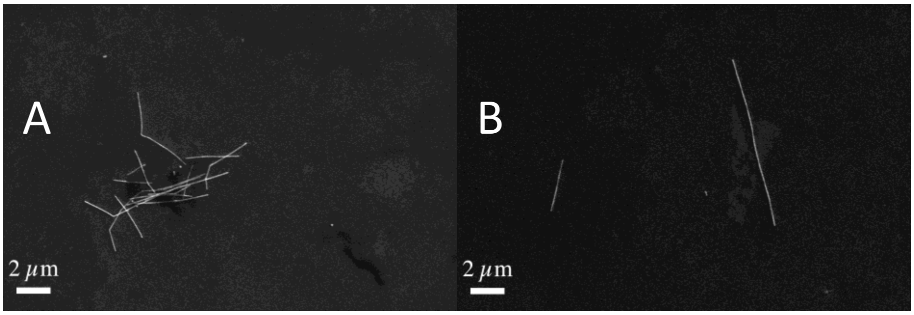

Supplemental **Figure S8. After one week in protein-rich media, Ag NW agglomerates (A) are detectable, but individual NWs (B) are still present.** Scanning electron microscopy (SEM) observations of S-Ag NWs following one week in aerobic cell culture medium (complete Dulbecco's modified eagle media containing 10% fetal calf serum (FCS).

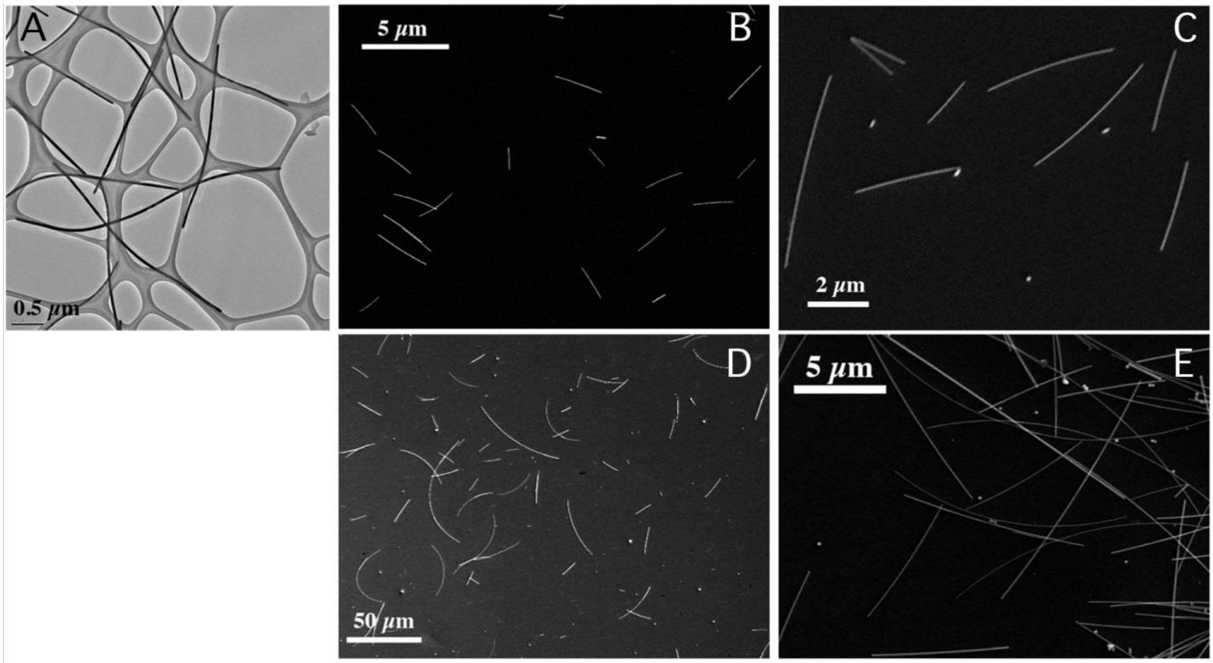

Supplemental Figure S9. Transmission electron microscopy (TEM) and scanning electron microscopy (SEM) characterization of PVP-coated Ag NWs after receipt from nanoComposix, Inc. and following different periods in storage. A. TEM micrograph of stock S-Ag NWs diluted in ultrapure water and deposited onto a lacey carbon grid. (TEM imaging of L-Ag NWs was not performed.) **B**. SEM micrograph of stock S-Ag NWs diluted in ultrapure water and immediately deposited on a cleaned silicon wafer. **C**. SEM image of S-Ag NWs that had been diluted in air-saturated ultrapure water and kept in the dark for two weeks. **D**. SEM micrograph of stock L-Ag NWs diluted in ultrapure water and immediately deposited on a cleaned silicon wafer. **E.** SEM image of L-Ag NWs that had been shipped to UC-Davis for instillation tests and returned to LBNL for characterization.
